# Fluctuations in Evolutionary Integration Allow for Big Brains and Disparate Faces

**DOI:** 10.1038/srep40431

**Published:** 2017-01-16

**Authors:** Kory M. Evans, Brandon T. Waltz, Victor A. Tagliacollo, Brian L. Sidlauskas, James S. Albert

**Affiliations:** 1 University of Louisiana at Lafayette, Department of Biology, P.O. Box 42451, Lafayette, LA, 70504, USA; 2 Universidade Federal do Tocantins, Programa de Pós-graduação Ciências do Ambiente (CIAMB), Palmas, Tocantins, 77001–090, Brazil; 3 Oregon State University, Department of Fisheries and Wildlife, 104 Nash Hall, Corvallis, OR, 97331, USA

## Abstract

In theory, evolutionary modularity allows anatomical structures to respond differently to selective regimes, thus promoting morphological diversification. These differences can then influence the rate and direction of phenotypic evolution among structures. Here we use geometric morphometrics and phenotypic matrix statistics to compare rates of craniofacial evolution and estimate evolvability in the face and braincase modules of a clade of teleost fishes (Gymnotiformes) and a clade of mammals (Carnivora), both of which exhibit substantial craniofacial diversity. We find that the face and braincase regions of both clades display different degrees of integration. We find that the face and braincase evolve at similar rates in Gymnotiformes and the reverse in Carnivora with the braincase evolving twice as fast as the face. Estimates of evolvability and constraints in these modules suggest differential responses to selection arising from fluctuations in phylogenetic integration, thus influencing differential rates of skull-shape evolution in these two clades.

The covariation between biological structures in evolution and in development has played a key role in structuring the phenotypic diversity of living and extinct organisms^1^,^2^,^3^. In theory, high levels of covariation among structures (i.e. integration) can constrain the range of producible phenotypes, as a result of a highly integrated pleiotropic network that slows the rate of evolution within these structures. Low levels of covariation relative to self -similarity (i.e. modularity) are posited to have the opposite effect, facilitating the evolution of functional specialization by relaxing the effects of an integrated pleiotropic network, thus allowing different modules to respond independently to selective forces^4^,^5^. This hypotheses was supported by a simulation study on mammal skulls by Marroig, Shirai^4^. In this study, clades with high integration exhibited a limited response to selection due to pleiotropic networks, integrating changes among associated parts and globalizing the effects of mutations. This integrated network was found to limit the capacity of the system as a whole to respond to selection. Contrariwise, clades with more modular phenotypes demonstrated more lability to selective forces.

The study of modularity in macroevolution has gained traction in recent years as investigators measure evolutionary and developmental covariation among embryologically or functionally defined modules^6^,^7^,^8^. The vertebrate skull has become a favorite model of evolutionary modularity and integration^9^,^10^, perhaps because of its remarkable diversification and specialization in every major vertebrate lineage^11^. The skull also performs a wide range of functions, including protecting and supporting the brain, sensory organs and cranial nerves, and other tissues involved in respiration, feeding and communication^12^,^13^. Within the skull, studies have frequently identified two distinct and partially-decoupled functional-anatomical regions (modules); the face and braincase, though other smaller modules have also been recovered^8^,^14^,^15^,^16^.

Here we examine two phylogenetically distant case studies in vertebrate skull evolution and describe the evolutionary consequences of modularity and integration between the face and braincase in terms of evolvability (ability to respond to selection), constraints, and rates of evolution. The examples are radiations of Neotropical electric fishes (Gymnotiformes, Teleostei) and Carnivora (Mammalia), both of which exhibit substantial diversity in craniofacial phenotypes, have similar phylogenetic ages (5-7E7 years), and similar species richness values (2-3E2 species)^17^,^18^,^19^,^20^,^21^.

We use 2-dimensional geometric morphometrics and the novel approach of Denton and Adams^22^ to quantify rates of face and braincase evolution within Gymnotiformes and Carnivora. Gymnotiformes range from brachycephalic (with a relatively large braincase and foreshortened snout) taxa to dolichocephalic (with a relatively small braincase and an elongated snout) taxa with many species exhibiting intermediate phenotypes^18^. Carnivorans range from brachycephalic mustelid species to dolichocephalic canids, and exhibit diverse ecologies associated with these divergent craniofacial phenotypes^21^,^23^ and rapid brain-size evolution^24^. In both gymnotiforms and carnivorans, these divergent craniofacial phenotypes may be the result of rapid responses to selective forces on the face and braincase modules. Here, we estimate evolvability and constraints for the face and braincase skull region in both clades using phenotypic matrix correlations^4^,^25^. We then use these methods to track the evolution of craniofacial shapes that characterize these clades.

We predict to find that the face exhibits faster rates of evolution than the braincase for Gymnotiformes as a result of the diverse craniofacial phenotypes and their functions exhibited within this clade^18^,^26^,^27^. We also predict higher rates of braincase evolution (as compared to facial evolution) within Carnivora as a result of the rapid brain size evolution that characterizes this clade^28^, and slower rates of facial evolution as a result of functional constraints related to feeding^29^. Additionally, we predict that the face will exhibit a stronger response to selection than the braincase in Gymnotiformes and that the opposite pattern will be found in Carnivora as a result of the observed phenotypic diversity associated with both modules in these clades.

## Results

### Craniofacial Diversity in Gymnotiformes

**Figure 1:**
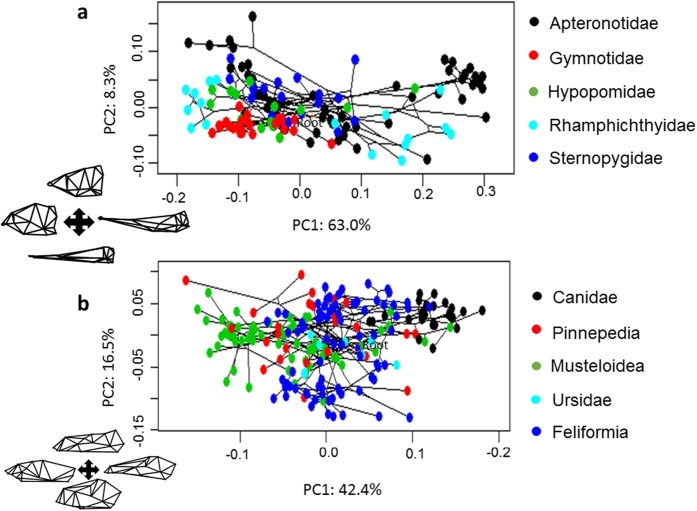
Phylomorphospace analyses of craniofacial shape in lateral view. (**a**) Phylomorphospace analysis of 133 species of Gymnotiformes (Teleostei), including all 35 recognized genera, with family-level clades delimited by colors. PC1 corresponds to variance between brachycephalic and dolichocephalic skull shapes, PC2 corresponds to variance in skull depth ranging from deep skulls with high values (*Adontosternarchus balaenops*) to narrow skulls with low values (*Orthosternarchus tamandua*). (**b**) Phylomorphospace analysis of 203 species of Carnivora (Mammalia) with clades delimited by colors. PC1 corresponds to variance along the brachycephalic to dolichocephalic axis, and PC2 corresponds to variance in skull depth.

**Figure 2:**
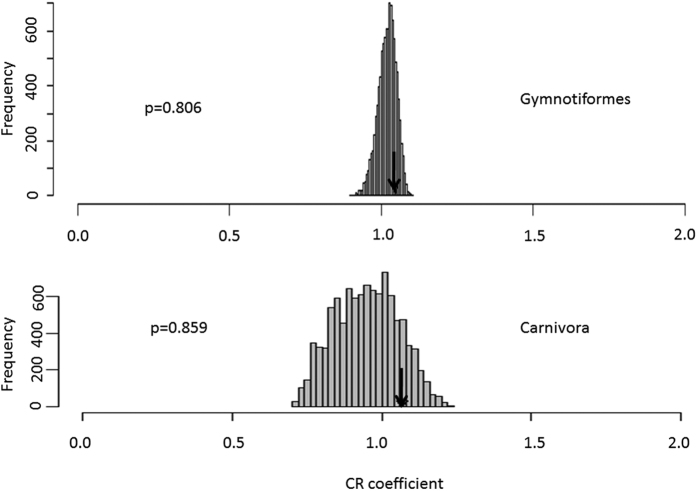
Phylogenetic modularity analysis of face and braincase regions for Gymnotiformes and Carnivora quantified using the covariance ratio (CR) coefficient. Note face and braincase modules were not found to exhibit significant degrees of modularity for either clade.

Gymnotiformes display a wide diversity of craniofacial phenotypes (Fig. 1a). Here, the PC1 axis describes variance between foreshortened (negative values) and elongate skull shapes (positive values). The PC2 axis corresponds to variance in skull depth, with positive values corresponding to deeper skulls (Fig. 2a,b).

Species of the family Apteronotidae have colonized nearly every portion of the empirical morphospace occupied by the other four gymnotiform clades. Apteronotids also possess the most extreme PC1 & PC2 values, with *Adontosternarchus* possessing the deepest skulls (highest PC2 values) and *Parapteronotus* and *Sternarchorhynchus*, the most elongate skulls (highest PC1 values). Rhamphichthyidae has a similar total spread of craniofacial disparity, although including only species with very foreshortened skulls (e.g. *Hypopygus* and *Steatogenys*) and very elongate skulls (e.g. *Gymnorhamphichthys* and *Rhamphichthys*) with no intermediate phenotypes. Gymnotidae occupies a distinct and confined portion of the observed morphospace, otherwise only occupied by the brachycephalic species of other families with the most foreshortened and slender skulls. Sternopygidae has a relatively conserved spread in morphospace, with a single colonization of a unique portion of the empirical morphospace (i.e. *Archolaemus blax*). On average, the sternopygid skull superficially resembles the intermediate craniofacial phenotypes of many apteronotid species (e.g. *Sternarchella orthos*). Hypopomidae follows a similar pattern, with a single lineage (*Akawaio*); occupying a unique portion of the observed morphospace. Most hypopomids possess a relatively foreshortened skull shape (e.g. *Brachyhypopomus*) although elongated skulls have evolved independently in *Akawaio* and *Hypopomus*.

### Craniofacial Diversity in Carnivora

Similarly to Gymnotiformes, PC1 of the carnivoran phylomorphospace (Fig. 1b) illustrates skull shape variance along a foreshortened (negative values) to elongated (positive values) axis. PC2 corresponds to skull depth with deep skulls occupying lower values and narrower skulls occupying higher values (Fig. 1b).

Along PC1, canids consistently possess the most dolichocephalic carnivoran skulls, with *Canis simensis* exhibiting the most dolichocephalic skull of all sampled carnivorans. Unlike canids, pinnipeds did not cluster in one particular region of the empirical morphospace. Instead, pinnipeds exhibit both the most brachycephalic (*Ommatophoca rossi*) and the shallowest (*Hydrurga leptonyx*) skulls of any sampled carnivoran. Feliformia do not occupy any extremes along the PC1 axis, however, they also exhibit a wide range of phenotypes along the PC2 axis with *Uncia uncia* possessing the deepest skull of any sampled carnivoran. Musteloids vary little in skull depth (PC2) yet vary widely along PC1 axis, and include the second-most brachycephalic carnivoran (*Aonyx capensis*). Ursids occupied various intermediate phenotypes.

### Phylogenetic Analysis of Modularity

An analysis of phylogenetic modularity using the covariance ratio coefficient found no significant signals of modularity between the face and braincase regions of gymnotiforms and carnivorans when compared to a Brownian motion model of evolution (Fig. 2).

### Phylogenetic Analysis of Integration

**Figure 3:**
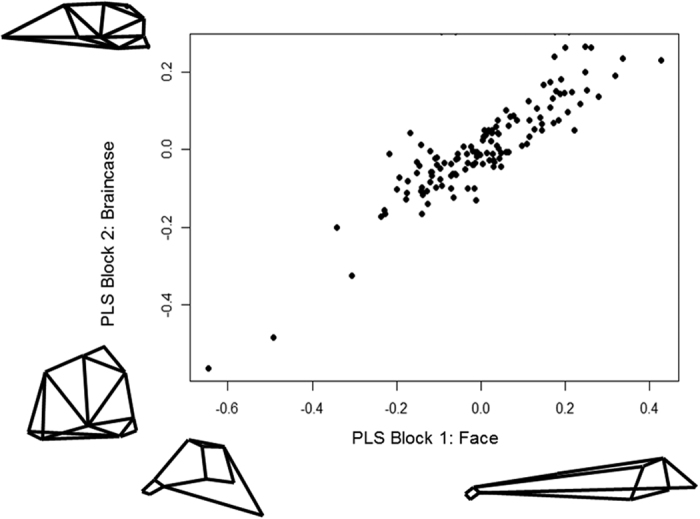
Phylogenetic Partial-Least Squares (PPLS) analysis of the face and braincase modules (blocks) of 133 species of Gymnotiformes. Note the strong but not complete pattern of covariation between the face and braincase modules (c = 0.905). Insets depict extreme patterns of deformation for each module.

**Figure 4:**
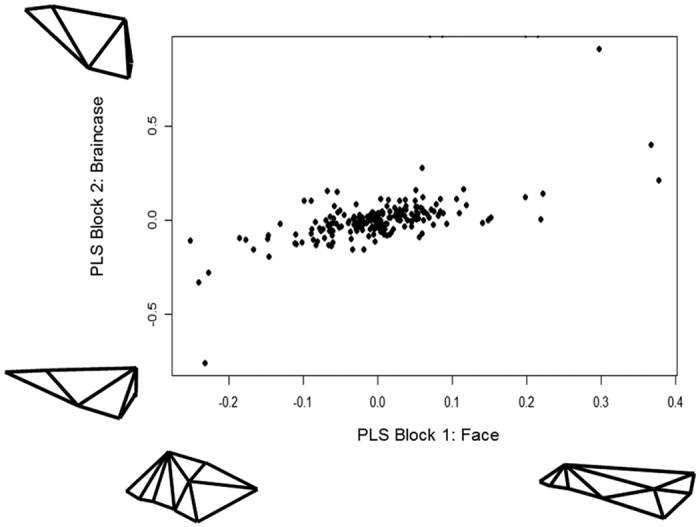
Phylogenetic Partial-Least Squares (PPLS) analysis of the face and braincase for 203 species of Carnivora. (Note) the weaker patter of integration (c = 0.627) between modules compared to Gymnotiformes). Insets depict extreme patterns of deformation for each module.

A phylogenetic partial-least squares analysis for Gymnotiformes (Fig. 3) indicated strong evolutionary integration between the face and braincase modules (correlation coefficient of 0.905, p = 9.9e-7). Within Carnivora, the analysis returned a significant (p = 0.017) but weaker correlation coefficient of 0.657, indicating significant, but weaker phylogenetic integration between craniofacial modules than in Gymnotiformes (Fig. 4).

### Rates of Craniofacial Evolution Gymnotiformes

**Figure 5:**
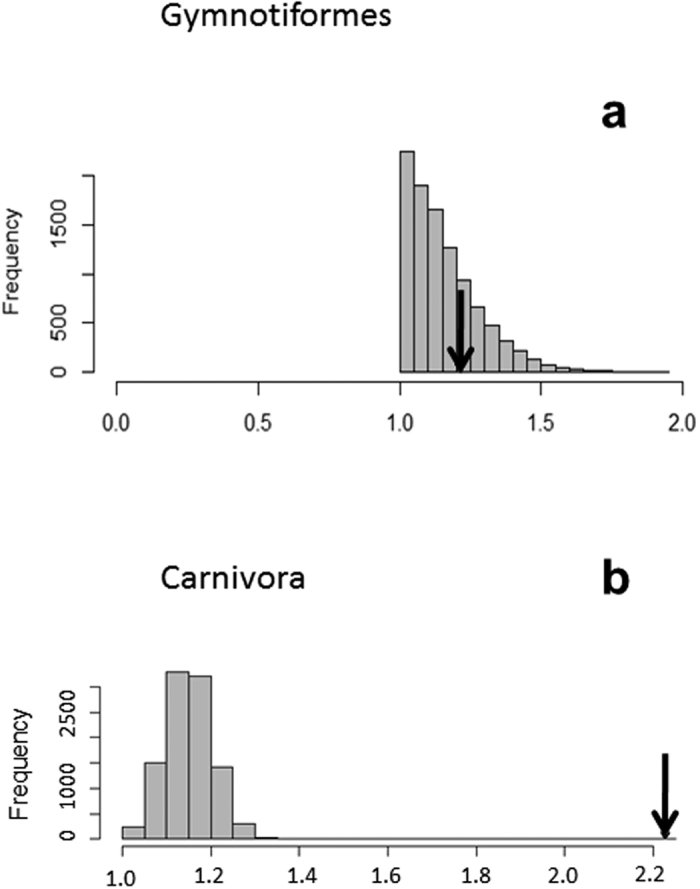
Histograms showing the ratio of rate values for module evolution for (**a**) Gymnotiformes and (**b**) Carnivora. Note in Carnivora, that the rate ratio falls outside the range of expected variation in values. Indicating significant differences in rates of evolution between face and braincase modules. However, in Gymnotiformes, no significant differences in rates between modules was recovered.

Within Gymnotiformes, the face and braincase modules were not found to evolve at significantly different rates (p = 0.271) (Fig. 5a) (Table 1). Among clades however, Apteronotidae, Gymnotidae and Hypopomidae exhibited the fastest rates of facial evolution (Table 1). There were no significant differences in rates among the three aforementioned clades, although each of the three fastest clades differed significantly from the slower Rhamphichthyidae and Sternopygidae (Supplementary Table 1). Sternopygidae exhibited the slowest rates of facial evolution, which was expected given their long branch-lengths and conserved distribution in the phylomorphospace. Interestingly, despite the high degree of morphological disparity in the Rhamphichthyidae, this clade returned the second-slowest rate of facial evolution in the analysis, likely due to long branch-lengths within this clade.

Apteronotidae and Gymnotidae exhibited the fastest rates of braincase evolution followed by Hypopomidae (Table 1). Rates of braincase evolution differed significantly between Hypopomidae, Apteronotidae and Gymnotidae (Supplementary Table 1). Rhamphichthyidae and Sternopygidae exhibited the slowest rates of braincase evolution. While all shape rate ratios were greater than 1.0, the facial module of Hypopomidae evolved about twice as fast as the braincase module, returning a ratio greater than 2.0.

### Rates of Craniofacial Evolution Carnivora

Rates of face and braincase evolution differed significantly within Carnivora (p = 0.0001) (Fig. 5b), with the braincase evolving twice as fast as the face. Feliformia exhibited the fastest rates of facial evolution, followed by Pinnepedia. Canidae, Musteloidea and Ursidae possessed the slowest rates of facial evolution (Table 1); pairwise p-value comparisons of rates among clades can be found in Supplementary Table 1.

Rates of shape evolution for the braincase were higher than in the face for all carnivore clades (Table 1). Feliformia exhibited substantially higher rates of braincase evolution than Canidae and Pinnepedia, followed by Musteloidea and Ursidae.

### Selection Simulations

Simulation studies suggests that the face and braincase modules display similar responses to selection vectors in Gymnotiformes, while the reverse holds true for Carnivora. Definitions for evolvability indices can be found in materials and methods. The face and braincase modules within Gymnotiformes show very similar responses to simulated selection vectors (Table 2), likely due to the high degree of integration between the modules. However, the face and braincase differ by an order of magnitude in conditional evolvability (ability of a clade to evolve in the direction of selection in the presence of integration) with the face exhibiting higher maximum and average values than the braincase. Additionally, the face and braincase differ by two orders of magnitude in autonomy (amount of evolvability that remains after conditioning on other traits) with the face exhibiting higher minimum, average and maximum values. These results suggest that the face has a better ability to respond to selective pressures while under the influence of conditioning on other traits via integration than the braincase in Gymnotiformes.

The braincase of carnivorans exhibits higher maximum values of unconditional evolvability (ability of a clade to evolve in the direction of selection) and respondability (how rapidly a clade responds to directional selection) than the face (Table 2), suggesting that the braincase structure is more evolvable than the face and has the ability to elicit a stronger response to selection than the face. The face exhibits a higher maximum value of autonomy than the braincase, suggesting that the face has a higher proportion of evolvability that remains after conditioning on other traits via integration.

## Discussion

### Evolutionary Disintegration Allows for Evolvability

While the face and braincase regions of gymnotiforms and carnivorans were not found to exhibit significant degrees of phylogenetic modularity; varying degrees of evolutionary integration between the face and braincase have left distinct signatures on the craniofacial diversity of both Carnivora and Gymnotiformes. The variation in evolutionary integration within these two clades has constrained how different modules of their skull respond to selective forces. In other words, fluctuations in the evolutionary lability within these skull modules allows for stronger responses to selection, leading to faster rates of skull evolution. Here we find that carnivoran skull exhibits modular evolution between the face and braincase regions. We also find that the braincase of carnivorans exhibits higher evolvability and respondability than the face of carnivorans. We hypothesize that this relaxed pattern of integration allowed for the braincase to respond to the strong selective pressures exerted on it by the brain during development in order to track the rapid brain size evolution that characterizes Carnivora and ultimately evolve at twice the rate of the face.

Within Gymnotiformes, the face and braincase modules were substantially more integrated. As a result, both modules elicited similar responses to selection pressures and appeared to be under similar influences of constraint. However, we find that the face exhibits higher conditional evolvability and autonomy than the braincase. Despite these differences in conditional evolvability and autonomy, no significant differences were found between the rates of face and braincase evolution.

### Rate Ratios Result from Selection and not Constraints

Within Gymnotiformes, we estimated constraints within face and braincase modules and found only slight differences between the maximum values (Table 2), suggesting that the face and braincase of Gymnotiformes are exposed to similar constraints. We interpret these results as selection for integration between face and braincase modules within Gymnotiformes. The source of this strong pattern of integration may also originate in the developmental pathway that forms the face. During development, *Shh* and *fgf8* signaling from the forebrain prompt the expansion of the face in development^30^,^31^,^32^,^33^,^34^. When this signaling is perturbed, the face fails to expand completely, resulting in a foreshortened facial phenotype. This signaling pattern transverses the face and braincase modules and can result in strong patterns of covariation between the face and braincase modules that can be conserved at the macroevolutionary scale^3^.

Within Carnivora, the face may be under strong functional constraints related to feeding and rotational torsion related to bite force. Christiansen and Wroe^35^ noted a trend towards increased bite force that has characterized specialization on larger prey and herbivory. The maintenance of these highly specialized morphologies requires tightly integrated morphogenetic programs, particularly those underlying the formation of teeth and tooth-bearing bones^36^. Among hyper carnivorous canid species, felids and mustelids, species that preyed on larger prey items all exhibit higher bite forces on the canine teeth than the carnassial teeth despite vastly different methods of prey acquisition. Furthermore, in all sampled carnivoran taxa (excluding hyaenids), bite forces at the canine and carnassial teeth exhibited little variation^35^. In a separate study by Goswami^15^ the anterior oral-nasal module which includes the anterior dentition and facial skeleton were found to be tightly integrated. This high integration between parts is expected in a functionally constrained system. When constraints were estimated between craniofacial modules of Carnivora, only slight differences between maximum values were recovered similar to the analysis in Gymnotiformes (Table 2). These results suggest that the face and braincase are also under similar constraints within Carnivora. We interpret these results as evidence for the role of selection on brain size in driving the higher rates of braincase evolution within Carnivora. During development, the braincase closely tracks the underlying neural tissue (brain) that it envelopes, such that if the brain were to undergo rapid size and shape evolution, the braincase would be expected to track it closely^37^,^38^. The factors that influence the rapid brain size evolution of carnivorans are less clear. It was initially believed that this rapid evolution coincided with the evolution of sociality within this clade in a hypothesis called the social-brain hypothesis (SBH)^39^,^40^. However, a more recent study by Finarelli and Flynn^24^ incorporated fossil taxa in their analysis and found no relationship between brain size and sociality.

Changes in the degree of integration appear to have had marked effects on the distribution of selective forces between the face and braincase for both Gymnotiformes and Carnivora. These differences in the distribution of selective forces allowed for more rapid rates of braincase evolution in Carnivorans and similar rates of module evolution in Gymnotiformes. This mosaic of selective forces and the resulting evolutionary responses that are elicited, have certainly influenced the evolution of the craniofacial shape diversity in both Gymnotiformes and Carnivora.

## Materials and Methods

### Gymnotiform Time Calibrated Phylogeny

In order to study the evolution of gymnotiform skulls, we used the phylogenetic hypothesis of Tagliacollo, Bernt^41^ trimmed to include only taxa examined for this study of craniofacial morphology (Supplementary Fig. 2). This phylogeny was obtained using a super-matrix comprised of six genes (5,054 bp), including three mitochondrial (16S, CytB, COI) and three nuclear (RAG1, RAG2, ZIC 1) markers, and 223 morphological characters (for tip taxa where molecular data was unavailable) concatenated using the Mkv model for morphology^42^ for 212 gymnotiform species representing 34 out of 35 extant genera^41^.

Lineage divergence times were estimated in *BEAST v 1*.*7*.*5*^43^ based on the Maximum Clade Credibility phylogeny of ostariophysan electric fishes (MCC-Gymn) inferred in *MrBayes v3*.*2*^44^ using the combining molecular + morphological datasets. Clade age estimation followed the approach of Tagliacollo *et al*.^45^ and employed identical data partitioning, evolutionary models used in, and geologic calibrations of the lognormal relaxed molecular clock. The prior constraints were based on fission-track age estimates for the initial rise of the Colombian Eastern Cordillera hypothesized to have isolated multiple cis and trans-Andean river basins at c. 11 Ma^46^. Divergence time estimates were composed of two independent Markov Chain Monte Carlo (MCMC) runs, each comprised of 5.0 × 107 generations. Parameter values were sampled every 5.0 × 103 generations, assuming the MCC-Gymnotiform phylogeny as the start tree, and a birth-death process for estimates of branching rates^47^. MCMC runs were combined using *LogCombiner* v1.7.5. All parameter estimates were inspected for stationary convergence prior to the burn-in procedure.

### Carnivora Time Calibrated Phylogeny

The comparative analysis of Carnivora trimmed the time-calibrated super tree of Nyakatura and Bininda-Emonds^19^ to include only taxa examined for this study of craniofacial morphology (Fig. S3).

### Specimen Preparation Gymnotiformes

We examined the neurocrania of 154 specimens representing 133 gymnotiform species (61% taxon sampling) (Supplementary Table 4), including all families and genera within Gymnotiformes, using 2-dimensional geometric morphometrics. Specimens were cleared and stained following the method of Taylor and Van Dyke^48^, and dissected under an Olympus SZX-12 stereomicroscope. After dissection, neurocrania were placed in a clay mold for stability and photographed in lateral view using a Nikon Coolpix digital camera. Specimens unavailable for clearing and staining were radiographed at the Academy of Natural Sciences in Philadelphia and Louisiana State University. The laterally compressed body-shape of most gymnotiform species allow specimens to be radiographed with little to no rotational effects.

### Specimen Preparation Carnivora

We examined the neurocrania of 445 specimens representing 203 carnivoran species (71% taxon sampling) (Supplementary Table 5), spanning all families using 2-dimensional geometric morphometrics. Adult specimen photos in lateral view were compiled from the online museum databases: DigiMorph (University of Texas, USA), Mammalian Crania Photographic Archive (Dokkyo Medical University, Japan), Museum Victoria (Australia), Animal Diversity Web (University of Michigan, USA), P. W. Lund’s collection (Natural History Museum of Denmark), as well as private collections and species descriptions. Specimens that were obviously rotated or damaged were removed from the analysis and only adult (as evidenced by fully erupted dentition) male specimens were analyzed to correct for potential secondary sexual-dimorphism and ontogenetic changes.

### Geometric Morphometrics

**Figure 6:**
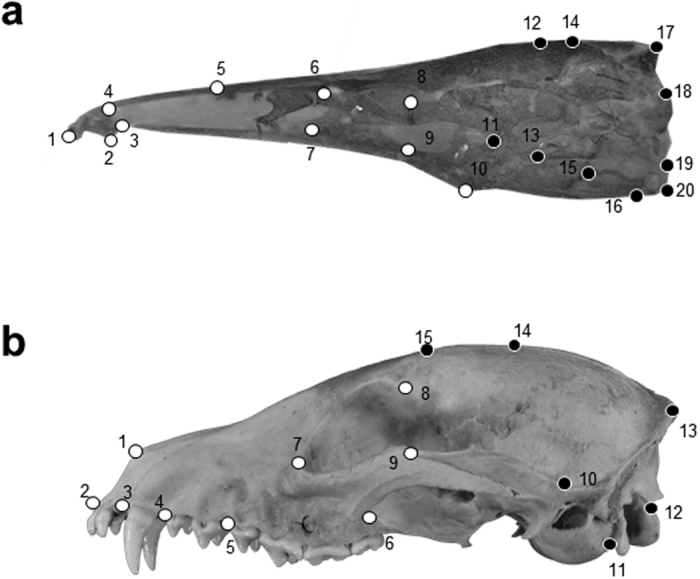
Landmark schematic of the neurocranium for gymnotiform and carnivoran clades used in the analysis. (**a**) Landmark schematic of *Compsaraia compsa* in lateral view Landmarks (n = 20) used in geometric morphometric analyses of gymnotiform fishes. (**b**) Landmark schematic of the neurocranium of *Cercodon thous* in lateral view showing landmarks (n = 15) used in geometric morphometric analyses of Carnivora. White landmarks indicate facial module landmarks and black landmarks indicate braincase module landmarks.

For Gymnotiformes, images were imported as tps files using *tpsUtil* and digitized with 20 homologous landmarks (Fig. 6a) (Supplementary Table 6) in *tpsDig2*. Tps files were then imported into *MorphoJ*^49^ and the R-package *Geomorph*^50^ for further statistical analyses. Procrustes superimposition removed the effect of size and orientation on the specimens and translated the landmark data to a common coordinate plane. The procure for Carnivora was similar, but used 15 landmarks in lateral view following the scheme of Figueirido, Tseng^23^ for the upper jaw (Fig. 6b).

Following Procrustes superimposition, principal components analyses based on covariance matrices summarized the variation in skull shape. For both clades, only the first two principal components were retained for subsequent analyses as they accounted for a large portion of the variance in each clade (Fig. S7).

### Phylomorphospace Analysis

We generated phylomorphospaces for Gymnotiformes and Carnivora in the R-package *phytools* by projecting the phylogenies into spaces defined by the first two principal components and estimating the position of internal nodes using maximum-likelihood^51^. Species nodes were color-coded by clade to allow for ease of interpretation.

### Phylogenetic Covariation

Phylogenetic covariation between the two craniofacial modules was quantified using two metrics to test for phylogenetic modularity and integration. Phylogenetic modularity was quantified using the covariance ratio (CR) coefficient performed in the *R-package Geomorph*^52^. In this analysis, the degree of phylogenetic modularity between the face and braincase modules was quantified under a Brownian motion model of evolution. Significance was assessed by randomly assigning landmarks into different subsets via permutation (9999 iterations) and comparing the observed CR value to the randomly distributed values. A significant signal of modularity was found when the observed CR coefficient was small relative to the random distribution. Phylogenetic integration was quantified using a phylogenetically-sensitive modification of the within-configuration partial-least squares analysis performed in the *R*-package *Geomorph*^50^. A partial-least squares analysis evaluates covariation between matrices (modules) and quantifies the relationship using a correlation coefficient. The correlation coefficient can be interpreted as 0 (completely modular) and 1 (completely integrated). In addition to the correlation coefficient, the statistical significance of the observed coefficient was computed by permutation (9999 iterations). Here the data from one PLS-block (module) was permuted across the tips of the tree to calculate an estimate of covariation in the two datasets and compare them to the observed correlation coefficient^53^.

### Module Designation Gymnotiformes

For the study of rates of module evolution, there were two hypothesized modules: the face (landmarks 1:10) and the braincase (landmarks 11:20) (Fig. 6b). These two modules were selected based on an *a priori* hypotheses of brain and skull development proposed in Albert^18^.

### Module Designation Carnivora

For the study of rates of module evolution within Carnivora, there were two hypothesized modules: the face (landmarks 1:9) and the braincase (landmarks 10:15). Modules were delimited following the conceptual scheme of Drake and Klingenberg^10^ (Fig. 6b). In previous studies conducted on the carnivoran skull, as many as six craniofacial modules have been hypothesized with varying degrees of within-module integration^54^. Here, our focus is on the face and braincase regions. As a result, other smaller modules have been pooled into these two larger modules. In our analysis, the facial region is a tightly integrated system^15^ that includes the anterior dentition and facial skeleton along with the posterior most dentition, the orbit and zygomatic arches. The braincase module consists of the basicranium and cranial vault modules as described by Goswami^15^.

### Module Evolution

Rates of evolution for the same face and braincase modules were estimated for five clades of Gymnotiformes and Carnivora in *Geomorph* using the “*compare. multi. rates*” function^22^. Clades were selected based on characteristic phenotypes and clade age. Significance was determined by comparing the observed rate ratios to a simulated null distribution of equal rates in both modules. The proportion of simulated ratios greater than or equal to the observed values, were treated as the significance level for each observed rate ratio.

### Evolutionary Simulations

Evolvability indexes of the face and braincase were estimated for both clades to using phenotypic matrix statistics^4^ to test for different responses to selection between modules. Phenotype V/CV matrices (P-matrices) were built using *MorphoJ* for Gymnotiformes and Carnivora. Within *MorphoJ*, ancestral state reconstructions were performed on P-matrices to account for phylogenetic non-independence in both clades and reconstruct phenotypic changes at internal nodes following the procedure of Linde‐Medina, Boughner^25^. Shape coordinates were not corrected for allometric scaling as not to exclude valuable information on the potential line of least evolutionary resistance that is thought to orient with shape changes associated with size (PC1)^4^,^55^. Following the construction of the P-matrices, we used the framework of Hansen and Houle^56^ to estimate different evolvability indices. While the original framework of Hansen and Houle^56^ is based on genetic matrices (G-matrices), P-matrices can be substituted in place of G-matrices if there is enough similarity between the two. In mammals, a close similarity between G and P matrices was strongly evidenced in Marroig, Shirai^4^ and in fishes, several studies have found significant correlations between G and P matrices^57^,^58^,^59^. Evolvability was estimated using the random skewers method of Cheverud and Marroig^60^ based on the Lande^61^ equation (Δz = Gβ) where G is the genetic variance-covariance matrix and β is the selection gradient. Here β is simulated as 1000 random selection vectors generated under a Gaussian distribution. The simulated β was then applied to the P-matrix to generate 1000 response vectors (Δz) as outlined by Marroig, Shirai^4^. These response vectors index unconditional evolvability (ability of a clade to evolve in the direction of selection), respondability (how rapidly a clade can respond to directional selection), conditional evolvability (ability of a clade to evolve in the direction of selection while under stabilizing selection), constraints (the effect of PC1 on the response to selection) and autonomy (the proportion of evolvability that remains after conditioning on other traits). All simulations were run in the R package *EvolQG*^62^. Instead of emphasizing mean values of evolvability indexes, we emphasize the maximum values of the various indexes for ease of interpretation^4^.

## Additional Information

**How to cite this article:** Evans, K. M. *et al*. Fluctuations in Evolutionary Integration Allow for Big Brains and Disparate Faces. *Sci. Rep.* **7**, 40431; doi: 10.1038/srep40431 (2017).

**Publisher's note:** Springer Nature remains neutral with regard to jurisdictional claims in published maps and institutional affiliations.

## Supplementary Material

Supplementary Information

Supplementary Dataset 1

Supplementary Dataset 2

Supplementary Dataset 3

Supplementary Dataset 4

Supplementary Dataset 5

Supplementary Dataset 6

Supplementary Dataset 7

Supplementary Dataset 8

Supplementary Dataset 9

Supplementary Dataset 10

Supplementary Dataset 11

Supplementary Dataset 12

## Figures and Tables

**Table 1:** Rates of module evolution and sigma-D ratios for Gymnotiformes and Carnivora.

Clade	Face σ	Braincase σ	σ Ratio
Gymnotiformes	0.0032	0.0027	1.2
Apteronotidae	0.0040	0.0036	1.13
Hypopomidae	0.0039	0.0019	2.03
Gymnotidae	0.0035	0.0031	1.13
Rhamphichthyidae	0.0023	0.0015	1.48
Sternopygidae	0.1340	0.0013	1.06
Carnivora	0.0022	0.0049	0.45
Canidae	0.0018	0.0046	0.40
Pinnepedia	0.0023	0.0035	0.65
Musteloidea	0.0014	0.0023	0.61
Feliformia	0.0029	0.0076	0.38
Ursidae	0.0014	0.0019	0.74

Note the similar rates of facial evolution with respect to braincase in gymnotiform clades. Note also the consistently faster rates of braincase evolution compared to facial evolution in carnivorans.

**Table 2:** Evolvability indexes for the selection simulation study for the face and braincase of Gymnotiformes and Carnivora.

Face					
Gymnotiformes	evolvability	respondability	conditional evolvability	autonomy	constraints
Mean	1.406E-04	2.445E-04	−5.712E-05	−0.593	0.556
Min	2.447E-05	6.522E-05	−0.061	−622.472	0.000
Max	4.065E-04	5.119E-04	0.003	23.721	0.997
**Braincase**					
Mean	1.751E-04	2.706E-04	−3.488E-06	−0.021	0.526
Min	4.998E-05	9.056E-05	−0.002	−10.484	0.001
Max	4.050E-04	5.433E-04	1.320E-04	0.647	0.978
**Face**					
**Carnivora**	**evolvability**	**respondability**	**conditional evolvability**	**autonomy**	**constraints**
Mean	1.745E-04	2.619E-04	−1.113E-06	−0.005	0.464
Min	3.780E-05	9.759E-05	−5.788E-04	−2.613	4.935E-04
Max	5.435E-04	5.762E-04	1.756E-04	1.208	0.977
**Braincase**					
Mean	4.338E-04	8.002E-04	−1.688E-06	−0.006	0.459
Min	3.303E-05	1.082E-04	−0.001	−3.212	0.001
Max	0.002	0.003	4.678E-04	0.744	0.970

Note the similarity between evolvability, respondability and constraints between the face and braincase of Gymnotiformes and the vast difference in maximum values maximum values of conditional evolvability and autonomy with the face exhibiting substantially higher values than the braincase. Note also within Carnivora, the high evolvability and respondability of the braincase compared to the face. In both Gymnotiformes and Carnivora the face exhibits higher conditional evolvability and higher autonomy than the braincase.
