# Supplementary material for: Fluctuations in Evolutionary Integration Allow for Big Brains and Disparate Faces
Source: Sci Rep. 2017 Jan 16;7:40431. doi: 10.1038/srep40431 (PMC5238424; doi:10.1038/srep40431)
Supplement: Supplementary Information [file srep40431-s1.pdf]

Fluctuations in Evolutionary Integration Allow for Big Brains and Disparate Faces

Kory M. Evans[1], Brandon T. Waltz[1], Victor A. Tagliacollo[2], Brian L. Sidlauskas[3] & James S. Albert[1]

[1] University of Louisiana at Lafayette, Department of Biology, P.O. Box 42451, Lafayette, LA 70504, USA. kxe9300@louisiana.edu (KME), btw6589@louisiana.edu (BTW),

jalbert@louisiana.edu (JSA)

[2] Universidade Estadual Paulista, Júlio de Mesquita Filho, Câmpus de Botucatu, Botucatu, Brazil. victor_tagliacollo@yahoo.com.br (VAT)

[3]Oregon State University, Department of Fisheries and Wildlife, 104 Nash Hall

Corvallis, OR 97331, USA. brian.sidlauskas@oregonstate.edu

Supplementary Table 1. Pairwise significance values for clade-specific analysis of rates of module evolution for face (above diagonal) and braincase (below diagonal) for Gymnotiformes and Carnivora tested against the null hypothesis of no difference in rate values (bold denotes significance).

**Gymnotiformes-Clade**	Apteronotidae	Gymnotidae	Hypopomidae	Sternopygidae	Rhamphichthyidae
Apteronotidae	-	0.319	0.761	0.001	0.001
Gymnotidae	0.297	-	0.614	0.001	0.039
Hypopomidae	0.001	0.004	-	0.001	0.019
Sternopygidae	0.001	0.001	0.027	-	0.013
Rhamphichthyidae	0.001	0.001	0.244	0.284	-
**Carnivora-Clade**	Canidae	Pinnepedia	Musteloidea	Feliformia	Ursidae
Canidae	-	0.132	0.025	0.001	0.156
Pinnepedia	0.13	-	0.001	0.041	0.013
Musteloidea	0.001	0.004	-	0.001	0.947
Feliformia	0.002	0.001	0.001	-	0.001
Ursidae	0.001	0.015	0.394	0.001	-

Supplementary Material 2. Phylogeny of 133 gymnotiform species used in analysis of neurocranial evolution. Images of neurocrania representing species used in morphometric analysis, in five family-level clades; Gymnotidae, Hypopomidae, Rhamphichthyidae, Sternopygidae and Apteronotidae. Time-calibrated tree from Tagliacollo et al. (2015; 2016).

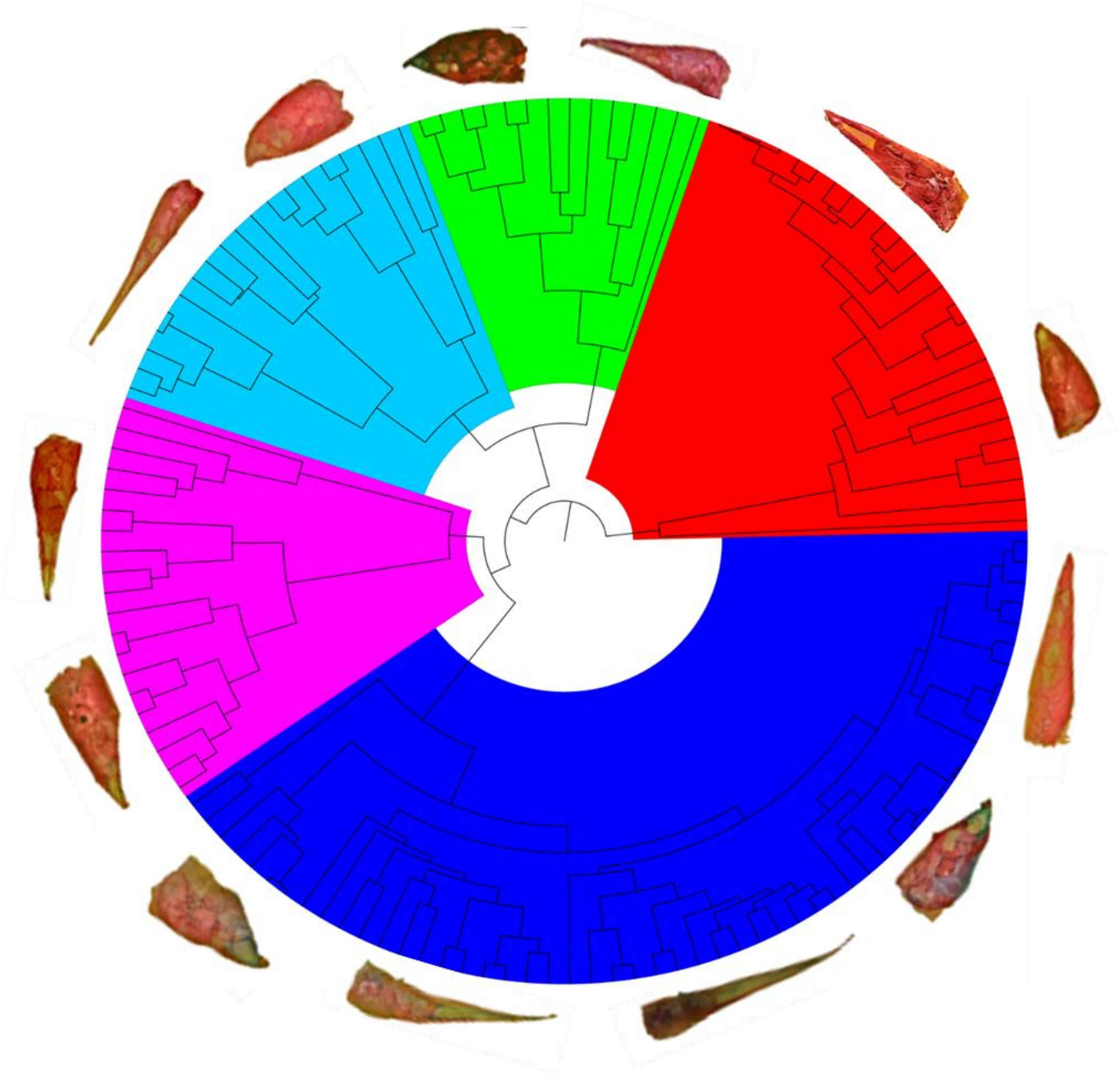

Supplementary Material 3. Phylogeny of 203 carnivoran species used in analysis of neurocranial evolution. Images of neurocrania representing species used in morphometric analysis, in five family-level clades; Feliformia, Canidae, Ursidae, Pinnepedia and Musteloidea. Time-calibrated tree from Nyakatura and Bininda-Emonds (2012).

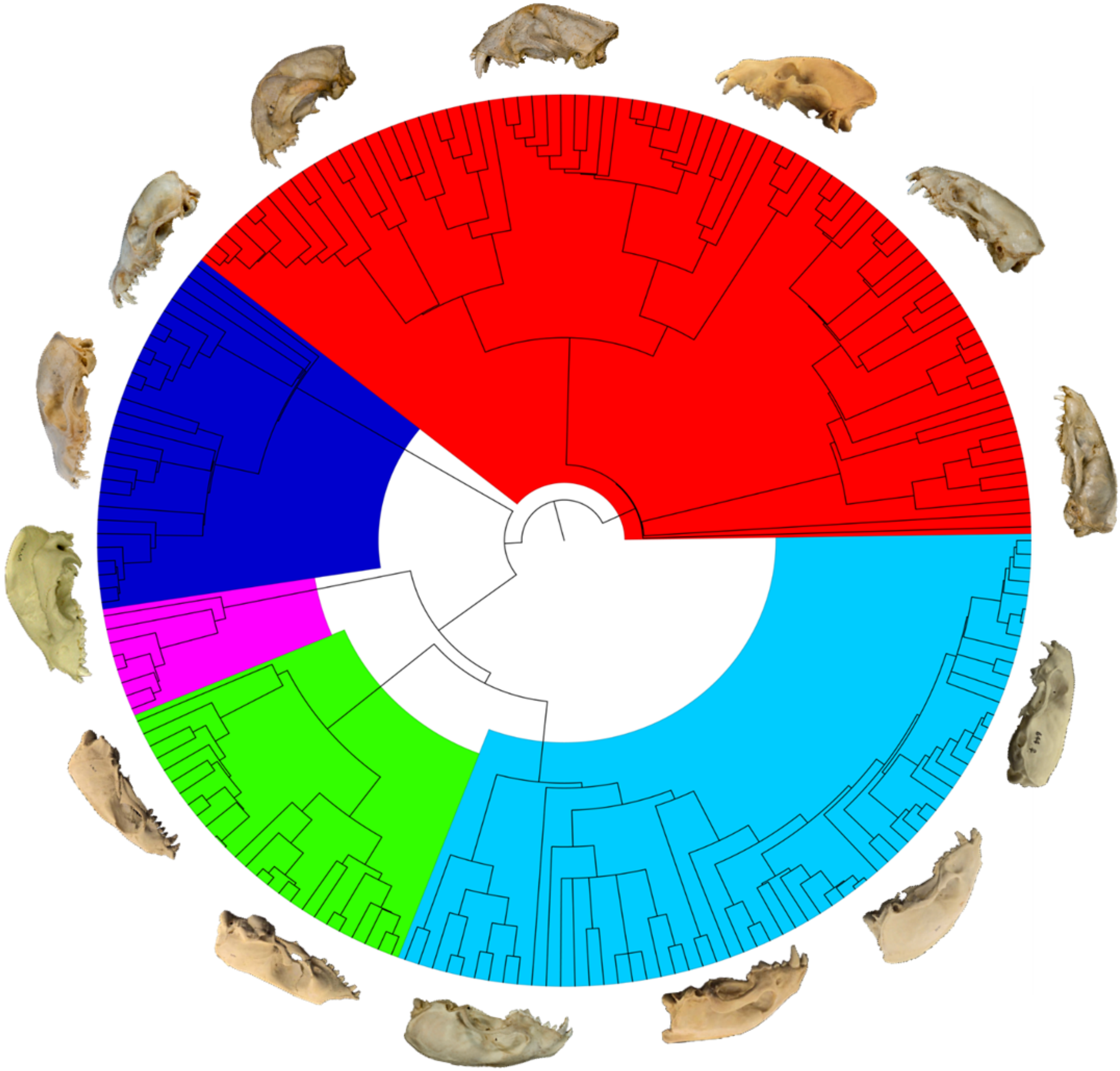

Supplementary Table 4. Specimens and museum catalog (Catalog #) information for each individual used for the interspecific study of gymnotiform neurocranial morphologies. Uncatalogued specimens (uncat) are accompanied by field numbers.

Species	Catalog #	Species	Catalog #	
Adontosternarchus balaenops	ANSP uncat. FD2015	*Gymnotus ucamara*	ANSP uncat. FD2015	
Adontosternarchus clarkae	FMNH 114766	*Gymnotus varzea*	ANSP uncat. FD2015	
Adontosternarchus devenanzii	ANSP uncat. FD2015	*Hypopomus artedi*	AUM 62945	
Adontosternarchus nebulosus	FMNH 117280	*Hypopygus cryptogenys*	INPA 29446	
Adontosternarchus sachsi	ANSP uncat. FD2015	*Hypopygus lepturus*	ANSP uncat. FD2015	
Akawaio penak	ROM 93463	*Hypopygus minissimus*	UF 148533	
Apteronotus albifrons	UF 29921	*Hypopygus neblinae*	ANSP uncat. FD2015	
Apteronotus apurensis	UMMZ 228869	*Hypopygus nijsseni*	ANSP uncat. FD2015	
Apteronotus baniwa	AUM 54987	*Hypopygus ortegai*	UF 148584	
Apteronotus bonapartii	ANSP uncat. FD2015	*Iracema caiana*	ANSP uncat. FD2015	
Apteronotus brasiliensis	ANSP 69647	*Magosternarchus duccis*	ANSP 192995	
Apteronotus cuchillejo	UF25559	*Magosternarchus raptor*	UF 116762	
Apteronotus cuchillo	UMMZ 157673	*Megadontognathus kaitukaensis*	ANSP 195961	
Apteronotus eschmeyeri	CAS 72115	*Microsternarchus bilineatus*	UNCAT	
Apteronotus leptorhynchus	UMMZ 145761	*Orthosternarchus tamandua*	ANSP uncat. FD2015	
Apteronotus macrolepis	ANSP uncat. MJG	*Parapteronotus hasemani*	CAS 56510	
Apteronotus macrostomus	FMNH 92638	*Pariosternarchus amazonensis*	MCP34917	
Apteronotus magdalenensis	USNM 123795	*Pariosternarchus amazonensis*	ANSP191996	
Apteronotus pemon	UF 33905	*Platyurosternarchus macrostomus*	ANSP uncat. FD2015	
Archolaemus blax	INPA 18451	*Porotergus duende*	ANSP uncat. FD2015	
Archolaemus janeae	ANSP 197978	*Porotergus gimbelli*	ANSP uncat. FD2015	

Brachyhypopomus beebei	MUSM 39375	*Procerusternarchus pixuna*	INPA 29494	
Brachyhypopomus bennetti	ANSP 178397	*Racenisia fimbriipinna*	ANSP uncat. FD2015	
Brachyhypopomus bombilla	UF 183773	*Rhabdolichops caviceps*	ANSP uncat. FD2015	
Brachyhypopomus brevirostris	ANSP uncat. IQ2015	*Rhabdolichops lundbergi*	ANSP uncat. FD2015	
Brachyhypopomus draco	ANSP uncat. FD2015	*Rhabdolichops navalha*	ANSP uncat. FD2015	
Brachyhypopomus gauderio	ANSP uncat. FD2015	*Rhabdolichops nigrimans*	ANSP uncat. FD2015	
Brachyhypopomus occidentalis	USNM 293152	*Rhabdolichops troscheli*	ANSP 197642	
Brachyhypopomus pinnicaudatus	ANSP uncat. FD2015	*Rhamphichthys apurensis*	ANSP 162300	
Brachyhypopomus walteri	ANSP uncat. FD2015	*Rhamphichthys drepanium*	UF 78066	
Compsaraia compsus	ANSP 163033	*Rhamphichthys hahni*	ANSP uncat. FD2015	
Compsaraia samueli	ANSP uncat. FD2015	*Rhamphichthys marmoratus*	ANSP uncat. FD2015	
Distocyclus conirostris	ANSP uncat. FD2015	*Rhamphichthys rostratus*	INPA 17645	
Eigenmannia cf limbata	ANSP uncat. FD2015	*Steatogenys duidae*	ANSP uncat. FD2015	
Eigenmannia humboldtii	AUM 28753	*Steatogenys elegans*	ANSP uncat. FD2015	
Eigenmannia macrops	ANSP uncat. FD2015	*Sternarchella calhamazon*	ANSP uncat. FD2015	
Eigenmannia microstoma	MZUSP 24643	*Sternarchella orinoco*	USNM 228727	
Eigenmannia nigra	ANSP uncat. FD2015	*Sternarchella orinoco*	USNM 228727	
Eigenmannia vicentespelaea	LBP 15289	*Sternarchella orinoco*	USNM 228727	
Eigenmannia virencens	ANSP uncat. FD2015	*Sternarchella orinoco*	USNM 228727	
Electrophorus electricus	UMMZ 2044265	*Sternarchella orthos*	USNM 228725	
Gymnorhamphichthys bogardusi	ANSP uncat. FD2015	*Sternarchella orthos*	USNM 228871	
Gymnorhamphichthys britskii	ANSP uncat. FD2015	*Sternarchella orthos*	USNM 228725	
Gymnorhamphichthys hypostomus	ANSP uncat. FD2015	*Sternarchella schotti*	UF 116570	
Gymnorhamphichthys petiti	UF 19898	*Sternarchella sima*	ANSP197107	
Gymnorhamphichthys rondoni	AUM 44617	*Sternarchella sima*	USNM 373114	

Gymnotus arapaima	ANSP uncat. FD2015	*Sternarchella sima*	USNM 373114	
Gymnotus bahianus	MCP 18110	*Sternarchella sima*	USNM 373114	
Gymnotus carapo	ANSP uncat. FD2015	*Sternarchella terminalis*	FMNH 115241	
Gymnotus carapo	AUM 20624	*Sternarchella terminalis*	MUSM uncat. Goulding 401	
Gymnotus carapo	MZUSP 30006	*Sternarchella terminalis*	MUSM uncat Goulding 401	
Gymnotus carapo	ANSP uncat. FD2015	*Sternarchella terminalis*	ANSP uncat. FD2015	
Gymnotus carapo	UF 36597	*Sternarchogiton labiatus*	ANSP uncat. FD2015	
Gymnotus anguillaris	UMMZ 190413	*Sternarchogiton nattereri*	ANSP uncat. FD2015	
Gymnotus chaviro	MUSM 33714	*Sternarchogiton porcinum*	ANSP uncat. FD2015	
Gymnotus chimarrao	UFRGS 6775	*Sternarchogiton preto*	USNM 233393	
Gymnotus choco	NRM 27734	*Sternarchorhamphus muelleri*	FMNH 115426	
Gymnotus coropinae	ANSP uncat. FD2015	*Sternarchorhamphus muelleri*	USNM 228807	
Gymnotus curupira	ANSP uncat. FD2015	*Sternarchorhynchus curvirostris*	ANSP 192774	
Gymnotus cylindricus	UMMZ 193986	*Sternarchorhynchus galibi*	ANSP 187155	
Gymnotus diamantinensis	ANSP uncat. FD2015	*Sternarchorhynchus goeldii*	ANSP uncat. FD2015	
Gymnotus inaequilabiatus	UMMZ 207025	*Sternarchorhynchus hagedornae*	ANSP uncat. FD2015	
Gymnotus javari	UMMZ 224596	*Sternarchorhynchus higuchii*	ANSP 194950	
Gymnotus javari	ANSP uncat. FD2015	*Sternarchorhynchus inpai*	ANSP 196466	
Gymnotus jonasi	ANSP uncat. FD2015	*Sternarchorhynchus kokraimoro*	ANSP 196380	
Gymnotus maculosus	ANSP uncat. FD2015	*Sternarchorhynchus montanus*	ANSP uncat. FD2015	
Gymnotus mamiraua	ANSP uncat. FD2015	*Sternarchorhynchus mormyrus*	ANSP 195483	
Gymnotus obscurus	ANSP uncat. FD2015	*Sternarchorhynchus oxyrhynchus*	USNM 228987	
Gymnotus obscurus	ANSP uncat. FD2015	*Sternarchorhynchus retzeri*	ANSP uncat. FD2015	
Gymnotus panamensis	ANSP uncat. FD2015	*Sternarchorhynchus stewarti*	ANSP 187151	
Gymnotus pantanal	UMMZ 206080	*Sternarchorhynchus yepezi*	ANSP 165222	

Gymnotus pantherinus	MCP20666	*Sternopygus aequilabiatus*	ANSP uncat. FD2015	
Gymnotus pantherinus	LSP 0932	*Sternopygus astrabes*	ANSP uncat. FD2015	
Gymnotus sylvius	LGP 2338	*Sternopygus branco*	ANSP uncat. FD2015	
Gymnotus tigre	UF 122821	*Sternopygus macrurus*	ANSP uncat. FD2015	
Gymnotus tigre	UF 122821	*Sternopygus xingu*	UMMZ 228961	
Gymnotus tiquie	MZUSP 85002	*Tembeassu marauna*	MZUSP 48510	

**Supplementary Table 5.** Materials for interspecific study of carnivoran neurocranial morphologies	
**Species**	**Catalog #**
Acinonyx jubatus	FMNH 29635
Acinonyx jubatus	UMMZ 114800
Ailuropoda melanoleuca	UMMZ 715TC
Ailurus fulgens	ILF 0080
Ailurus fulgens	ILF 0075
Ailurus fulgens	UMMZ 114841
Aonyx capensis	ILF 0117
Aonyx capensis	ZMA.MAM.24687
Aonyx cinerea	AMNH 101460
Aonyx cinerea	ILF0063
Arctictis binturong	UMMZ 49642
Arctictis binturong	USNM 259101
Arctocephalus pusillus	NMV C5717
Arctocephalus pusillus	ILF 0011
Arctocephalus pusillus	ILF 0012
Arctocephalus pusillus	MV 29173
Arctocephalus gazella	Goswami et al., 2015
Arctocephalus tropicalis	MV 2422
Arctogalidia trivirgata	UMMZ 68709
Arctonyx collaris	MVZ 186562
Arctocephalus fosteri	MV 6190
Arctocephalus galapagoensis	Jeffeson et al., 1993
Arctocephalus townsendi	MVZ191003
Atelocynus microtis	UCMVZ 157978
Atilax paludinosus	UMMZ 98654
Bassaricyon alleni	FMNH 86908
Bassaricyon gabbii	AMNH 140334
Bassariscus astutus	UMMZ 63902

Bassariscus astutus	ILF 0049
Bassariscus astutus	ILF 0052
Bassariscus sumichrasti	UMMZ 114646
Bdeogale crassicauda	ROM 58389
Callorhinus ursinus	MVZ 175109
Canis aureus	UMMZ 101191
Canis latrans	UMMZ 82499
Canis latrans	ILF0056
Canis latrans	ILF0057
Canis latrans	WSP 800
Canis lupus	TMM M-1709
Canis lupus	ILF 0145
Canis lupus	UMMZ 60914
Canis lupus	WSP
Canis mesomelas	DKY 0147
Canis mesomelas	DKY 1744
Canis mesomelas	DKY 1874
Canis simensis	AMNH 81001
Caracal caracal	UMMZ 166222
Caracal caracal	ILF 0149
Catopuma temminckii	Pocock, 1939
Cerdocyon thous	UMMZ 126121
Cerdocyon thous	PWL 107
Chrotogale owstoni	UMMZ 32556
Chrysocyon brachyurus	UMMZ 170489
Civettictis civetta	UMMZ 161756
Civettictis civetta	ILF 0129
Conepatus chinga	UMMZ 124451
Conepatus humboldtii	UMMZ 157158
Conepatus leuconotus	UMMZ 63993
Conepatus semistriatus	UMMZ 80292
Crocuta crocuta	MVZ 184551
Crocuta crocuta	ILF 0151
Crossarchus alexandri	Sonet et al., 2014
Crossarchus ansorgei	Sonet et al., 2014
Crossarchus obscurus	Sonet et al., 2014
Crossarchus platycephalus	Sonet et al., 2014
Cryptoprocta ferox	UMMZ 33950
Cuon alpinus	Heptner et al., 1988
Cynictis penicillata	UMMZ 103553
Cynogale bennettii	UMMZ 145587

Cystophora cristata	MWNH 187
Diplogale hosei	UMMZ uncat
Eira barbara	UMMZ 126847
Enhydra lutris	UMMZ 103762
Enhydra lutris	ILF 0062
Enhydra lutris	WSP
Erignathus barbatus	UMMZ uncat
Eumetopias jubatus	UCMP 22889
Eupleres goudotii	UMMZ 100484
Felis catus	DKY 0132
Felis catus	DKY 0165
Felis catus	DKY 0466
Felis catus	DKY 0645
Felis catus	DKY 1091
Felis catus	THK 0340
Felis catus	UMMZ 88418
Felis chaus	UMMZ 122370
Felis manul	DKY 2662
Felis manul	DKY 2692
Felis manul	DKY 2693
Felis manul	Heptner et al., 1988
Felis margarita	UMMZ 118429
Felis nigripes	Renard et al., 2015
Felis silvestris	MSU 24293
Fossa fossana	UMMZ 100454
Galerella sanguinea	UMMZ 113416
Galictis cuja	UMMZ 174833
Galictis vittata	UMMZ 115521
Galidia elegans	UMMZ 318105
Genetta abyssinica	UMMZ 27218
Genetta angolensis	UMMZ 469853
Genetta genetta	MSU 24280
Genetta genetta	ILF0050
Genetta genetta	ILF0051
Genetta johnstoni	UMMZ uncat
Genetta maculata	UMMZ 467584
Genetta piscivora	UMMZ 13071
Genetta servalina	UMMZ 36017
Genetta thierryi	UMMZ 436342
Genetta tigrina	UMMZ 107925
Genetta victoriae	UMMZ 51420

Gulo gulo	USNM 314885
Gulo gulo	ILF 0058
Halichoerus grypus	WSP
Halichoerus grypus	SMNH 2880
Helarctos malayanus	FMNH 54316
Helogale parvula	MSU 11572
Hemigalus derbyanus	UMMZ 68725
Herpestes edwardsi	UMMZ 122377
Herpestes ichneumon	MSU 23982
Herpestes javanicus	UMMZ 75161
Hyaena hyaena	USNM 182034
Hyaena brunnea	ILF 0150
Hydrictis maculicollis	MWNH 343
Hydrurga leptonyx	USNM 270326
Ichneumia albicauda	UMMZ 107924
Ictonyx striatus	uncat
Ictonyx striatus	ILF 0125
Leopardus geoffroyi	DKY 2806
Leopardus geoffroyi	UMMZ 146504
Leopardus guigna	MSU 2116
Leopardus pardalis	DKY 2663
Leopardus pardalis	ILF 0064
Leopardus pardalis	UMMZ 79533
Leopardus tigrinus	UMMZ 64041
Leopardus wiedii	UMMZ 126123
Leptailurus serval	UMMZ 38386
Leptailurus serval	DKY 2674
Leptonychotes weddellii	NH 3637
Lobodon carcinophaga	UMMZ 82247
Lontra canadensis	UCLA 15275
Lontra felina	ROM 1299
Lontra provocax	Larviere, 1999
Lutra lutra	MWNH 414
Lutra lutra	WSP
Lutrogale perspicillata	FMNH 63799
Lycaon pictus	USNM 368441
Lycaon pictus	WSP
Lycalopex culpaeus	UMMZ 157157
Lycalopex griseus	UMMZ 165626
Lycalopex griseus	ILF 0140
Lycalopex sechurae	MUSM 2093

Lycalopex sechurae	ILF 0037
Lycalopex gymnocercus	UMMZ 124458
Lycalopex gymnocercus	DKY 0681
Lyncodon patagonicus	MNHN CG 1897-422
Lynx rufus	UCLA 10115
Lynx rufus	WSP
Macrogalidia musschenbroekii	UMMZ uncat
Martes americana	ILF 0031
Martes americana	ILF 0038
Martes americana	UMMZ 97784
Martes flavigula	SZMN 6869
Martes foina	SZMN 47409
Martes martes	WSP
Martes pennanti	ILF 0054
Martes pennanti	UMMZ 173984
Martes melampus	DKY 1109
Martes melampus	DKY 1111
Martes melampus	DKY 1133
Martes melampus	DKY 1159
Martes melampus	DKY 1188
Martes melampus	DKY 1223
Martes melampus	DKY 1226
Martes melampus	DKY 1332
Martes melampus	DKY 1333
Martes melampus	DKY 1372
Martes melampus	DKY 1373
Martes melampus	DKY 1374
Martes melampus	DKY 1375
Martes melampus	DKY 1376
Martes melampus	DKY 1377
Martes melampus	DKY 1425
Martes melampus	DKY 1427
Martes melampus	DKY 1431
Martes melampus	DKY 1435
Martes melampus	DKY 1454
Martes melampus	DKY 1455
Martes melampus	DKY 1456
Martes melampus	DKY 1490
Martes melampus	DKY 1491
Martes melampus	DKY 1492
Martes melampus	DKY 1916

Martes pennanti	ILF 0048
Martes zibellina	Monakhov, 2011
Meles meles	DKY 0008
Meles meles	DKY2234
Meles meles	DKY2584
Meles meles	DKY 2668
Meles meles	THK 0031
Meles meles	THK 0188
Meles meles	THK 0388
Meles meles	uncat
Mellivora capensis	MSU 8093
Melogale moschata	UMMZ 97616
Melogale personata	ZM HNU-M 776
Melursus ursinus	RAMM 5034651
Mephitis macroura	DKY 1681
Mephitis macroura	DKY 1783
Mephitis macroura	DKY 2144
Mephitis macroura	DKY 2216
Mephitis macroura	UMMZ 110950
Mephitis mephitis	DKY 1861
Mephitis mephitis	DKY 2680
Mephitis mephitis	DKY 2717
Mephitis mephitis	ILF 0039
Mephitis mephitis	ILF 0040
Mephitis mephitis	USNM 147553
Mirounga angustirostris	MVZ 184140
Monachus monachus	Scheel et al., 2014
Monachus tropicalis	USNM 100358
Mungos mungo	UMMZ 107926
Mustela africana	Ramirez-Chevez et al., 2015
Mustela erminea	UMMZ 86233
Mustela felipei	Ramírez-Chaves & Patternson, 2014
Mustela frenata	UMMZ 82577
Mustela itasi	DKY 0644
Mustela itasi	DKY 0948
Mustela itasi	DKY0950
Mustela itasi	DKY 0961
Mustela itasi	DKY 0963
Mustela itasi	DKY 1051

Mustela itasi	DKY 1078
Mustela itasi	DKY 1079
Mustela itasi	DKY 1107
Mustela itasi	DKY 1114
Mustela itasi	DKY 1121
Mustela itasi	DKY 1122
Mustela itasi	DKY 1139
Mustela itasi	DKY 1149
Mustela itasi	DKY 1150
Mustela itasi	DKY 1151
Mustela itasi	DKY 1218
Mustela itasi	DKY 1220
Mustela itasi	DKY 1323
Mustela itasi	DKY 1324
Mustela itasi	DKY 1336
Mustela itasi	DKY 1339
Mustela itasi	DKY 1462
Mustela itasi	DKY 1474
Mustela itasi	DKY 1494
Mustela itasi	DKY 1499
Mustela itasi	THK 0389
Mustela kathiah	UMMZ 112553
Mustela nigripes	UMMZ 103451
Mustela nivalis	ILF 0047
Mustela nivalis	uncat
Mustela lutreola	SZMN 6878
Mustela eversmanii	SZMN 6993
Mustela putorius	DKY 2222
Mustela putorius	UMMZ 98648
Mustela putorius	Heptner et al., 1988
Mydaus javanensis	AMNH 106635
Mydaus marchei	FMNH 62878
Nandinia binotata	DKY 2295
Nandinia binotata	USNM 450440
Nasua narica	UMMZ 83182
Nasua nasua	DKY 0458
Nasua nasua	DKY 1274
Nasua nasua	DKY 1996
Nasua nasua	DKY 2195
Nasua nasua	DKY 2672
Nasua nasua	UMMZ 124457

Nasuella olivacea	USNM 240034
Nasuella meridensis	USNM 143658
Neofelis diardi	RMNH 71/41
Neofelis nebulosa	ILF 0136
Neofelis nebulosa	ILF 0184
Neofelis nebulosa	USNM 282124
Neophoca cinerea	NSMT C 27557
Neovison vison	DKY 0345
Neovison vison	DKY 0346
Neovison vison	DKY 0347
Neovison vison	DKY 0351
Neovison vison	DKY 0352
Neovison vison	DKY 0355
Neovison vison	DKY 0359
Neovison vison	DKY 0361
Neovison vison	DKY 0362
Neovison vison	DKY 0363
Neovison vison	DKY 0364
Neovison vison	DKY 0365
Neovison vison	DKY 0366
Neovison vison	DKY 0368
Neovison vison	DKY 0369
Neovison vison	DKY 0370
Neovison vison	DKY 0371
Neovison vison	DKY 0373
Neovison vison	DKY 0375
Neovison vison	DKY 0376
Neovison vison	DKY 0377
Neovison vison	DKY 0379
Neovison vison	DKY 0386
Neovison vison	DKY 0393
Neovison vison	DKY 0394
Neovison vison	DKY 0395
Neovison vison	DKY 0396
Neovison vison	DKY 0398
Neovison vison	DKY 0399
Neovison vison	DKY 0401
Neovison vison	UMMZ 97999
Nyctereutes procyonoides	DKY 0334
Nyctereutes procyonoides	DKY 0735
Nyctereutes procyonoides	DKY 0736

Nyctereutes procyonoides	DKY 0738
Nyctereutes procyonoides	DKY 0739
Nyctereutes procyonoides	DKY 0939
Nyctereutes procyonoides	DKY 0952
Nyctereutes procyonoides	DKY 1017
Nyctereutes procyonoides	DKY 1018
Nyctereutes procyonoides	DKY 1050
Nyctereutes procyonoides	DKY 1054
Nyctereutes procyonoides	DKY 1055
Nyctereutes procyonoides	DKY 1063
Nyctereutes procyonoides	DKY 1067
Nyctereutes procyonoides	DKY 1080
Nyctereutes procyonoides	DKY 1088
Nyctereutes procyonoides	DKY 1089
Nyctereutes procyonoides	DKY 1129
Nyctereutes procyonoides	THK 0155
Nyctereutes procyonoides	USNM 255530
Odobenus rosmarus	MVZ 125566
Ommatophoca rossii	MACN-Ma 48.259
Otocyon megalotis	DKY 1745
Otocyon megalotis	DKY 1824
Otocyon megalotis	DKY 2386
Otocyon megalotis	DKY 2387
Otocyon megalotis	DKY 2444
Otocyon megalotis	DKY 2673
Otocyon megalotis	USNM 429129
Paguma larvata	DKY 0279
Paguma larvata	DKY 0650
Paguma larvata	DKY 1485
Paguma larvata	DKY 2394
Paguma larvata	DKY 2745
Paguma larvata	UMMZ 97621
Panthera leo	DKY 0926
Panthera leo	ILF 0007
Panthera leo	UMMZ 114804
Panthera onca	UMMZ 76743
Panthera pardus	DKY 2019
Panthera pardus	ILF 0159
Panthera pardus	ILF 0175
Panthera pardus	UMMZ 157858
Panthera tigris	DKY 2102

Panthera tigris	UMMZ 167640
Paracynictis selousi	MSU 8098
Paradoxurus hermaphroditus	DKY 2189
Paradoxurus hermaphroditus	UMMZ 85365
Phoca largha	DKY 1633
Phoca largha	NSMT uncat
Phoca vitulina	uncat
Phoca vitulina	ILF 0077
Phocarctos hookeri	OM VT087
Poecilogale albinucha	MNHN CG 1934-107
Poiana richardsonii	UMMZ uncat
Potos flavus	ILF 0154
Potos flavus	ILF 0158
Potos flavus	WSP
Potos flavus	UMMZ 114645
Prionailurus bengalensis	DKY 0704
Prionailurus bengalensis	DKY 0836
Prionailurus bengalensis	DKY 2665
Prionailurus bengalensis	DKY 2688
Prionailurus bengalensis	ILF 0076
Prionailurus bengalensis	AMNH 102458
Prionailurus iriomotensis	PRI 9001
Prionailurus planiceps	FMNH 60476
Prionailurus rubiginosus	FMNH 96335
Prionailurus viverrinus	DKY 2684
Prionailurus viverrinus	AMNH 101627
Prionodon linsang	USNM 303036
Procyon cancrivorus	ILF 0074
Procyon cancrivorus	UMMZ 146502
Procyon lotor	ILF 0164
Procyon lotor	UMMZ 98905
Procyon lotor	WSP
Procyon pygmaeus	Goldman & Jackson 1950
Proteles cristata	UMMZ 118657
Pteronura brasiliensis	UWSP
Puma concolor	WSP
Puma concolor	DKY 2202
Puma concolor	ILF 0148
Puma concolor	LACM 87430
Puma yagouaroundi	UMMZ 146505
Pusa hispida	UMMZ 55702

Rhynchogale melleri	uncat
Speothos venaticus	PWL 112M
Speothos venaticus	PWL 114M
Speothos venaticus	UMMZ 115805
Spilogale putorius	ILF 0060
Spilogale putorius	UMMZ 110951
Suricata suricatta	DKY 2182
Suricata suricatta	DKY 2363
Suricata suricatta	DKY 2579
Suricata suricatta	DKY 2586
Suricata suricatta	DKY 2593
Suricata suricatta	DKY 2626
Suricata suricatta	DKY 2784
Suricata suricatta	MVZ 118450
Taxidea taxus	UMMZ 77317
Taxidea taxus	WSP
Tremarctos ornatus	FMNH 41292
Uncia uncia	UMMZ 157859
Urocyon cinereoargenteus	WSP
Urocyon cinereoargenteus	ILF 0034
Urocyon cinereoargenteus	UCLA 6928
Ursus americanus	WSP
Ursus americanus	USNM 227070
Ursus arctos	WSP
Ursus arctos	TMM M-2749
Viverra tangalunga	DKY 2013
Viverra tangalunga	DKY 2014
Viverra tangalunga	UMMZ 85115
Viverra zibetha	UMMZ 60140
Viverricula indica	UMMZ 97619
Vormela peregusna	SZMN 2229
Vulpes bengalensis	UMMZ 75162
Vulpes chama	ILF 0036
Vulpes chama	MWNH 571
Vulpes corsac	UMMZ 118430
Vulpes lagopus	UMMZ 165969
Vulpes lagopus	ILF 0033
Vulpes lagopus	WSP
Vulpes lagopus	WSP
Vulpes macrotis	ILF 0035
Vulpes macrotis	WSP

Vulpes rueppellii	UMMZ 122374
Vulpes velox	UMMZ 88064
Vulpes vulpes	DKY 0153
Vulpes vulpes	DKY 0662
Vulpes vulpes	DKY 0663
Vulpes vulpes	DKY 0695
Vulpes vulpes	DKY 0811
Vulpes vulpes	DKY 0834
Vulpes vulpes	DKY 0835
Vulpes vulpes	DKY 0860
Vulpes vulpes	DKY 0881
Vulpes vulpes	DKY 0906
Vulpes vulpes	DKY 0912
Vulpes vulpes	DKY 0913
Vulpes vulpes	DKY 0920
Vulpes vulpes	DKY 0951
Vulpes vulpes	DKY 0962
Vulpes vulpes	DKY 0980
Vulpes vulpes	DKY 1013
Vulpes vulpes	DKY 1016
Vulpes vulpes	DKY 1070
Vulpes vulpes	DKY 1092
Vulpes vulpes	DKY 1100
Vulpes vulpes	UMMZ 52825
Vulpes zerda	ILF 0096
Vulpes zerda	UMMZ 159320
Vulpes zerda	WSP
Zalophus californianus	DKY 0094
Zalophus californianus	UCLA 252
Ursus maritimus	H 001-05

Supplementary Table 6. Landmark definitions for geometric morphometric analysis of Gymnotiformes and Carnivora

**Clade**	**LM #**	**Definition**
**Gymnotiformes**	1	Most anterior point of Mesethmoid
	2	Most anterior point of Ventral Ethmoid
	3	Posterior margin of Ventral Ethmoid and Mesethmoid
	4	Parasphenoid/Ventral Ethmoid suture
	5	Frontal/Mesethmoid suture
	6	Anterior Frontal/Orbitosphenoid suture

	7	Most anterior lower projection of Orbitosphenoid
	8	Orbitosphenoid/Pterosphenoid suture
	9	Posterior Orbitosphenoid/Parasphenoid suture
	10	Lower ridge of Parasphenoid
	11	Posterior Parasphenoid/Pterosphenoid suture
	12	Frontal/ Parietal suture
	13	Most anterior point of Prootic Foramen
	14	Supraoccipital/Parietal suture
	15	Basioccipital/Exoccipital/Prootic intersection
	16	Parasphenoid/Basioccipital suture
	17	Most superior inflection of Supraoccipital
	18	Supraoccipital/Exocciptial suture
	19	Exoccipital/Basioccipital suture
	20	Posterior corner of Basioccipital
**Carnivora**	1	Most anterior tip of nasal bones
	2	Most anterior point of premaxilla
	3	Frontal point in the sutura incisivomaxillaris at level of dentary row
	4	Point behind canine on dentary
	5	Point below the lacrimal foramen at level of dentary row
	6	Posterior point of dentary row
	7	Lacrimal foramen
	8	Tip of the supraorbital process
	9	Jugal/squamosal suture
	10	Tip of mastoid process
	11	Posterior point of typanic bulla
	12	Superior point of the occipital condyle
	13	Most posterior point of sagittal crest
	14	Intersection between sutura coronalis, sutura sagittalis, and sutura interfrontali
	15	Frontal/ Parietal suture

Supplementary Material 7. Percent variance of principal components for principal components analysis of Gymnotiformes (A) and Carnivora (B).

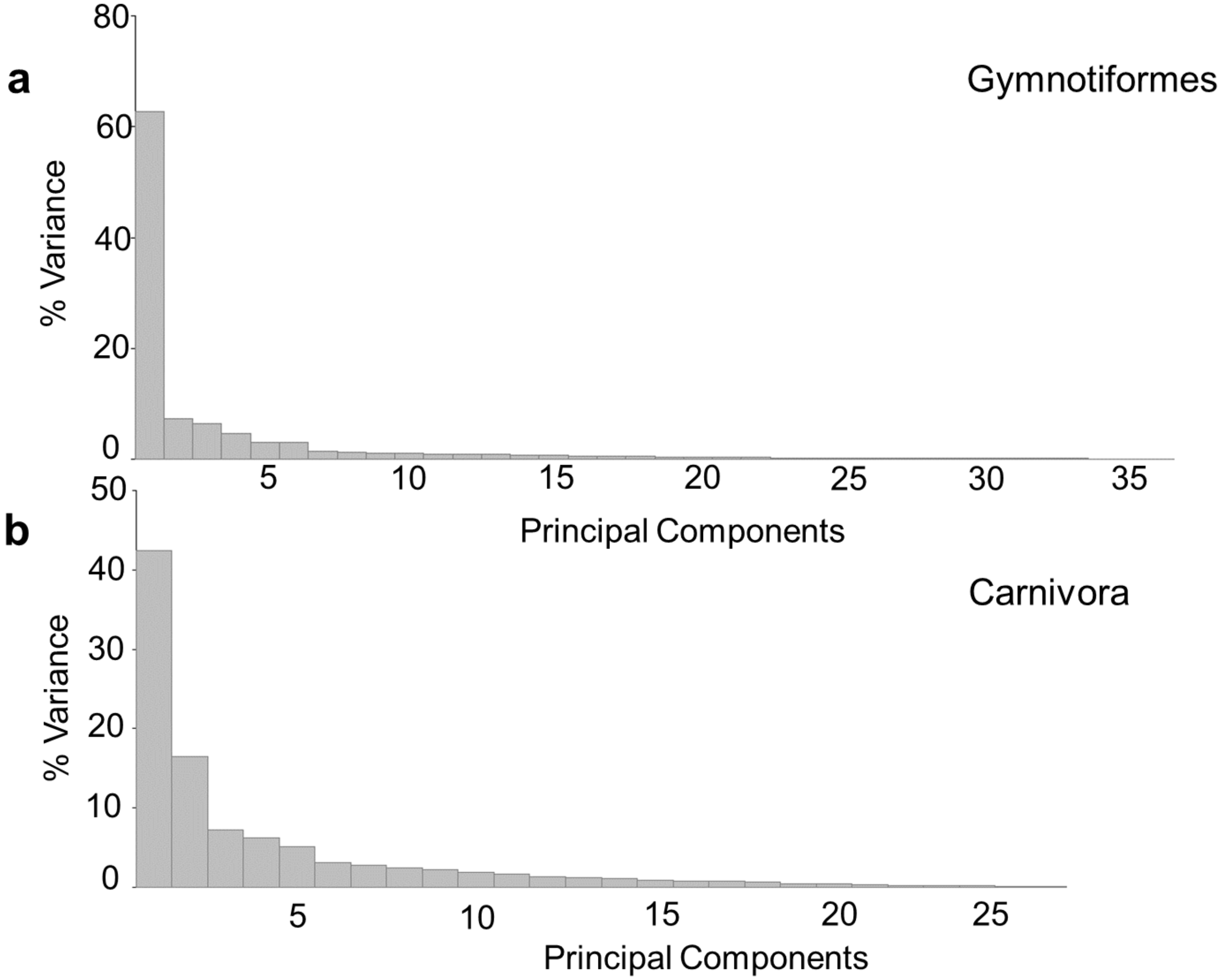

```
#########R-script for carnivoran analysis#####

require(ape)

require(phytools)

require(geiger)

require(geomorph)

par(mar=c(5,5,5,5))

#####Carnivoran Analyses####

Car_tree<-read.tree("carnivora_tee.txt")

plot(Car_tree,cex=0.5)

Car_data <-read.csv("Carnivorans.csv", row.names=1,header = T)

TreeOnly <- setdiff(Car_tree$tip.label,rownames(Car_data))

TreeOnly # Enter the name of the object we just created to see what's in it.

DataOnly <- setdiff(rownames(Car_data), Car_tree$tip.label)

DataOnly # Enter to see what species are in the data set but not the tree.

# In our case, we have overlap issues in both directions. Because we have data for fewer taxa than we have in our phylogeny, let's first prune our tree to just those species in the tree that were also measured before proceeding further.

# We'll prune the tree using drop.tip. We need to give it our tree, and a list of species to prune. We'll use the TreeOnly list of species names we just made to prune these species from the tree.


pruned_tree <- drop.tip(Car_tree,TreeOnly)

plot(pruned_tree)

phyloTime <- pruned_tree # Load a ultrametric tree

phyloTimeLadderized <- (ladderize(phyloTime))  # Ladderization

phyloTimeLadderized <- rescale(phyloTimeLadderized, "depth", 1) #This rescaling will make subsequent plotting functions somewhat easier. Even more importantly, it will often improve the performance of likelihood functions

plot(phyloTimeLadderized, cex=0.5, no.margin = T) #Plot ladderized and rescaled tree

add.scale.bar() # Add a simple scale bar indicating the scale for the branches in your tree
```

```
write.tree(phyloTimeLadderized, "phyloTimeLadderized.nwk")

phyloTimeLadderized <- read.tree("phyloTimeLadderized.nwk")

Ctree<- phyloTimeLadderized

tmp <- read.csv('Carnivorans.csv', header = TRUE, row.names =1,
        stringsAsFactors = FALSE)

shape <- as.matrix(sapply(tmp[,-(1:3)], as.numeric))

# here we say, use all columns except the first three.


is.numeric(shape)

[1] TRUE # now it's numeric. Ready to go!

#Gonna Need names and Classifiers

names <- tmp[,1]

coords <- arrayspecs(shape[,1:ncol(shape)], 15,2)

#arrayspecs- substitute the column number where the coordinates begin for the 2, p=number of
landmarks, k=number of dimensions

dimnames(coords)[[3]] <- names

#Procrustes Analysis

classifier <- read.csv('carnivora_classifier.csv',header = TRUE,row.names = 1, colClasses=c('factor',
'factor'))

#Then check to make sure your classifiers are factors...seriously! check!

is.factor(classifier$Family)

##assign groups to stuff

groups<-(classifier$Family)

names(groups) <- Ctree$tip.label

##Landmark Subsets for Modularity study

face<-coords[c(1:10),,]

braincase<-coords[c(11:15),,]

land.gp<-c("A","A","A","A","A","A","A","A","A","A","B","B","B","B","B")
```

```
EMR<-compare.multi.evol.rates(A=coords,gp=land.gp,
                        Subset=TRUE, phy= Ctree,iter=9999)
#####Phylogenetic Integration Test#####
IT<- phylo.integration(coords,partition.gp=land.gp,phy=Ctree,iter=9999)
summary(IT) # Test summary
plot(IT)
#####Phylogenetic Modularity Test#####
MT<-phylo.modularity(coords, partition.gp=land.gp, phy=Ctree, iter = 9999)
summary(MT)
plot(MT)
######Compare rates of module evolution among groups####
h1.1 <-compare.evol.rates(A=face, phy=Ctree,gp=groups,iter=9999)
h1.1sig1 <- h1.1$sigmad.all
h1.1sig2 <- h1.1$sigma.d
h1.2 <- compare.evol.rates(A=braincase, phy=Ctree,gp=groups,iter=9999)
h1.2sig1 <- h1.2$sigmad.all
h1.2sig2 <- h1.2$sigma.d
#######Phylomorphospace######
dataInput <- read.csv("Carnivora_PC.csv") #Load character data for Gymntotiformes
dataInput #Check out your data
workingData <- data.frame(dataInput[,2:4]) #Convert data in Data Frame in R
rownames(workingData) <- dataInput[,1]
attach(workingData)
name.check(Ctree, workingData)
head(workingData)

workingData <- workingData[Ctree$tip.label,] # sort our trait data to match the order of the tips in the tree
rownames(workingData) == Ctree$tip.label # check if it is sorted
```

Make a vector for PC scores

PC1 <- workingData[,1]

names(PC1) <- row.names(workingData)

PC2 <- workingData[,2]

names(PC2) <- row.names(workingData)

discTraitOne <- workingData[,3]

names(discTraitOne) <- row.names(workingData)

Phylomorphospace

Tip colors per family

tip.cols_cldA <- rep("black", 26)   # N of taxa

tip.cols_cldB <- rep("blue", 26)   # N of taxa

tip.cols_cldC <- rep("red", 63)   # N of taxa

tip.cols_cldd <- rep("green", 80)

tip.cols_clde <- rep("yellow", 8)

cols <- c(tip.cols_cldA, tip.cols_cldB, tip.cols_cldC,tip.cols_cldd,tip.cols_clde)

names(cols) <- 1:length(cols)

cols <- setNames(palette()[1:length(unique(discTraitOne))],sort(unique(discTraitOne)))

tipLabels <- character(length(Ctree$tip.label)) #We're going to make a new matrix to store the colors will use to label our tip taxa

names(tipLabels) <- 1:length(tipLabels)

tipLabels[discTraitOne=="1"] <- "black"

```
tipLabels[discTraitOne=="2"] <- "red"

tipLabels[discTraitOne=="3"] <- "green3"

tipLabels[discTraitOne=="4"] <- "blue"

tipLabels[discTraitOne=="5"] <- "cyan"



nodeLabels <- character(length(1:Ctree$Nnode)) #We're going to make a new matrix to store the colors will use to label our tip taxa

names(nodeLabels) <- 1:Ctree$Nnode



par(mar=c(5,5,5,5))

#plot phylomorphospace

phylomorphospace(Ctree, cbind(PC1,PC2), ylab = "PC2: Skull depth 13%", xlab = "PC1: Skull Length 31%", control=list(col.node=tipLabels), label = "OFF")

#######Evolvability Carnivora#####

require(evolvability)

G<-read.csv("CarnivoraCov.csv",row.names=1, header=T)

G<- as.matrix(G)

Beta<- randomBeta(1000,30)

EB<-evolvabilityBeta(G, Beta, means = 1)

##########Face####

G<-read.csv("FaceCov.csv",row.names=1, header=T)

G<- as.matrix(G)

Beta<- randomBeta(1000,18)

EBF<-evolvabilityBeta(G, Beta, means = 1)

#####Braincase#####

G<-read.csv("BraincaseCov.csv",row.names=1, header=T)

G<- as.matrix(G)
```

```
Beta<- randomBeta(1000,12)

EBB<-evolvabilityBeta(G, Beta, means = 1)


####### R-Script For gymnotiform analysis####

require(ape)

require(phytools)

require(geiger)

require(geomorph)

Gymn_tree<-read.tree("Full_Gymnotiform_tree.txt")

plot(Gymn_tree,cex=0.5)

Gymn_data <-read.csv("All_Gymn_proc_coords.csv", row.names=1)

TreeOnly <- setdiff(Gymn_tree$tip.label,rownames(Gymn_data))

TreeOnly # Enter the name of the object we just created to see what's in it.

DataOnly <- setdiff(rownames(Gymn_data), Gymn_tree$tip.label)

DataOnly # Enter to see what species are in the data set but not the tree.

# In our case, we have overlap issues in both directions. Because we have data for fewer taxa than we have in our phylogeny, let's first prune our tree to just those species in the tree that were also measured before proceeding further.

# We'll prune the tree using drop.tip. We need to give it our tree, and a list of species to prune. We'll use the TreeOnly list of species names we just made to prune these species from the tree.


pruned_tree <- drop.tip(Gymn_tree,TreeOnly)

plot(pruned_tree)

phyloTime <- pruned_tree # Load a ultrametric tree

phyloTimeLadderized <- (ladderize(phyloTime))  # Ladderization

phyloTimeLadderized <- rescale(phyloTimeLadderized, "depth", 1) #This rescaling will make subsequent plotting functions somewhat easier. Even more importantly, it will often improve the performance of likelihood functions

plot(phyloTimeLadderized, cex=0.5, no.margin = T) #Plot ladderized and rescaled tree

add.scale.bar() # Add a simple scale bar indicating the scale for the branches in your tree
```

```
write.tree(phyloTimeLadderized, "phyloTimeLadderized.nwk")

phyloTimeLadderized <- read.tree("phyloTimeLadderized.nwk")

tree<- phyloTimeLadderized

##########

tmp <- read.csv('All_Gymn_proc_coords.csv', header = TRUE, row.names =1,
        stringsAsFactors = FALSE)

shape <- as.matrix(sapply(tmp[,-(1:3)], as.numeric))

# here we say, use all columns except the first three.


is.numeric(shape)

[1] TRUE # now it's numeric. Ready to go!

#Gonna Need names and Classifiers

names <- tmp[,1]

coords <- arrayspecs(shape[,1:ncol(shape)], 20,2)

#arrayspecs- substitute the column number where the coordinates begin for the 2, p=number of
landmarks, k=number of dimensions

dimnames(coords)[[3]] <- names

#Procrustes Analysis

classifier <- read.csv('Morpho_classifierG.csv',header = TRUE,row.names = 1, colClasses=c('factor',
'factor'))

#Then check to make sure your classifiers are factors...seriously! check!

is.factor(classifier$Family)

##assign groups to stuff

groups<-(classifier$Family)

names(groups) <- tree$tip.label

face<-coords[c(1:10),,]

braincase<-coords[c(11:20),,]

land.gp<-c("A","A","A","A","A","A","A","A","A","A","B","B","B","B","B","B","B","B","B","B")
```

```
##########Compare Rates of Module Evolution#######

EMR<-compare.multi.evol.rates(A=coords,gp=land.gp,

                         Subset=TRUE, phy= tree,iter=9999)

#####Phylogenetic Modularity Test#####

MT<-phylo.modularity(coords, partition.gp=land.gp, phy=tree, iter = 9999)

summary(MT)

plot(MT)

#######Phylogenetic Integration Test######

IT<- phylo.integration(coords,partition.gp=land.gp,phy=tree,iter=9999)

summary(IT) # Test summary

plot(IT)

######Compare rates of module evolution among groups####

h1.1 <-compare.evol.rates(A=face, phy=tree,gp=groups,iter=9999)

h1.1sig1 <- h1.1$sigmad.all

h1.1sig2 <- h1.1$sigma.d

h1.2 <- compare.evol.rates(A=braincase, phy=tree,gp=groups,iter=999)

h1.2sig1 <- h1.2$sigmad.all

h1.2sig2 <- h1.2$sigma.d

########Phylomorphospace####

dataInput <- read.csv("All_gymn_skull_loadings.csv") #Load character data for Gymntotiformes

dataInput #Check out your data

workingData <- data.frame(dataInput[,2:4]) #Convert data in Data Frame in R

rownames(workingData) <- dataInput[,1]

attach(workingData)

name.check(phyloTimeLadderized, workingData)

head(workingData)


workingData <- workingData[phyloTimeLadderized$tip.label,] # sort our trait data to match the order of
the tips in the tree
```

```
rownames(workingData) == phyloTimeLadderized$tip.label # check if it is sorted



# Make a vector for PC scores
PC1 <- workingData[,1]
names(PC1) <- row.names(workingData)
PC2 <- workingData[,2]
names(PC2) <- row.names(workingData)
discTraitOne <- workingData[,3]
names(discTraitOne) <- row.names(workingData)



#### Phylomorphospace


Tip colors per family
tip.cols_cldA <- rep("black", 54)                         # N of taxa
tip.cols_cldB <- rep("blue", 26)                # N of taxa
tip.cols_cldC <- rep("red", 15)                 # N of taxa
tip.cols_cldd <- rep("green", 20)
tip.cols_clde <- rep("yellow", 18)
cols <- c(tip.cols_cldA, tip.cols_cldB, tip.cols_cldC,tip.cols_cldd,tip.cols_clde)
names(cols) <- 1:length(cols)

cols <- setNames(palette()[1:length(unique(discTraitOne))],sort(unique(discTraitOne)))

tipLabels <- character(length(phyloTimeLadderized$tip.label)) #We're going to make a new matrix to
store the colors will use to label our tip taxa
names(tipLabels) <- 1:length(tipLabels)
```

```
tipLabels[discTraitOne=="0"] <- "black"

tipLabels[discTraitOne=="1"] <- "red"

tipLabels[discTraitOne=="2"] <- "green3"

tipLabels[discTraitOne=="3"] <- "blue"

tipLabels[discTraitOne=="4"] <- "cyan"



nodeLabels <- character(length(1:phyloTimeLadderized$Nnode)) #We're going to make a new matrix to store the colors will use to label our tip taxa

names(nodeLabels) <- 1:phyloTimeLadderized$Nnode



par(mar=c(5,5,5,5))

#plot phylomorphospace

phylomorphospace(phyloTimeLadderized, cbind(PC1,PC2), ylab = "PC2: Skull depth 8.3%", xlab = "PC1: Skull Length 63%", control=list(col.node=tipLabels), label = "OFF")

#########Evolvability Gymnotiformes####

require(evolvability)

G<-read.csv("GymnCov.csv",row.names=1, header=T)

G<- as.matrix(G)

Beta<- randomBeta(1000,40)

EBF<-evolvabilityBeta(G, Beta, means = 1)

##########Face####

G<-read.csv("FaceCovG.csv",row.names=1, header=T)

G<- as.matrix(G)

Beta<- randomBeta(1000,20)

EBF<-evolvabilityBeta(G, Beta, means = 1)

#####Braincase#####

G<-read.csv("BrancaseCovG.csv",row.names=1, header=T)
```

G<- as.matrix(G)

Beta<- randomBeta(1000,20)

EBB<-evolvabilityBeta(G, Beta, means = 1)

Carnivoran Phylogeny

((Homo_sapiens:0.7810951936,(Rattus_norvegicus:0.2302064567,Mus_musculus:0.2302064567)3:0.5508887369)2:0.2189048064,(((Bos_taurus:0.4893775864,Equus_caballus:0.4893775864)6:0.4497773596,(((((((((((Acinonyx_jubatus:0.1081004968,(Puma_concolor:0.07306624682,Puma_yagouaroundi:0.07306624682)18:0.03503425001)17:0.1160899063,(((Lynx_canadensis:0.04677556983,Lynx_lynx:0.04677556983)21:0.004708672509,Lynx_pardinus:0.05148424234)20:0.04519870446,Lynx_rufus:0.09668294679)19:0.1275074563)16:0.01445746953,(((((((Felis_bieti:0.03096412447,Felis_silvestris:0.03096412447)28:0.01466937726,Felis_catus:0.04563350173)27:0.02944616666,Felis_margarita:0.07507966839)26:0.02654618628,Felis_chaus:0.1016258547)25:0.003998865674,Felis_nigripes:0.1056247204)24:0.127210028,((((Prionailurus_bengalensis:0.04541034785,Prionailurus_iriomotensis:0.04541034785)32:0.009418805911,Prionailurus_viverrinus:0.05482915376)31:0.06210649279,Prionailurus_planiceps:0.1169356465)30:0.00424423099,Prionailurus_rubiginosus:0.1211798775)29:0.1116548708)23:0.001983206192,Felis_manul:0.2348179545)22:0.003829918133)15:0.06980476379,((((Leopardus_braccatus:0.06358846143,Leopardus_colocolo:0.06358846143)36:0.04096903851,(Leopardus_geoffroyi:0.04853680414,(Leopardus_guigna:0.03999738822,Leopardus_tigrinus:0.03999738822)38:0.008539415924)37:0.0560206958,Leopardus_pajeros:0.1045574999)35:0.0185092111,(Leopardus_pardalis:0.0573198876,Leopardus_wiedii:0.0573198876)39:0.06574682345)34:0.01808623421,Leopardus_jacobitus:0.1411529453)33:0.1672996912)14:0.004584914734,((Catopuma_badia:0.1316652211,Catopuma_temminckii:0.1316652211)41:0.03308144728,Pardofelis_marmorata:0.1647466684)40:0.1482908828)13:0.0314762193,((Caracal_caracal:0.1033144073,Profelis_aurata:0.1033144073)43:0.08624587384,Leptailurus_serval:0.1895602811)42:0.1549534893)12:0.009531223021,(Neofelis_nebulosa:0.2212508418,(((Panthera_leo:0.07461044388,Panthera_pardus:0.07461044388)47:0.0265548992,Panthera_onca:0.1011653431)46:0.05315241909,(Panthera_tigris:0.09039154689,Uncia_uncia:0.09039154689)48:0.06392621529)45:0.0669330796)44:0.1327941517)11:0.3979814557,(Prionodon_linsang:0.1824027875,Prionodon_pardicolor:0.1824027875)49:0.5696236617)10:0.000854664108,(((((Arctictis_binturong:0.1640550319,((Macrogalidia_musschenbroekii:0.1087140014,(Paradoxurus_hermaphroditus:0.08251094784,(Paradoxurus_jerdoni:0.04924102237,Paradoxurus_zeylonensis:0.04924102237)58:0.03326992548)57:0.02620305357)56:0.00165621574,Paguma_larvata:0.1103702172)55:0.05368481474)54:0.07075920897,((Chrotogale_owstoni:0.1008750894,(Diplogale_hosei:0.06077920909,Hemigalus_derbyanus:0.06077920909)61:0.04009588026)60:0.03611991835,Cynogale_bennettii:0.1369950077)59:0.09781923317)53:0.03267307029,Arctogalidia_trivirgata:0.2674873112)52:0.1040446399,(((Civettictis_civetta:0.1500915692,(((Viverra_civettina:0.0397623557,Viverra_megaspila:0.0397623557)67:0.003721029588,Viverra_zibetha:0.04348338529)66:0.01999826263,Viverra_tangalunga:0.06348164792)65:0.08660992126)64:0.03869304205,Viverricula_indica:0.1887846112)63:0.1799478174,(((Genetta_abyssinica:0.06429719837,Genetta_thierryi:0.06429719837)70:0.1080645935,(((((((Genetta_angolensis:0.02354022569,Genetta_pardina:0.02354022569)78:0.002802099461,(Genetta_bourloni:0.009539985641,Genetta_poensis:0.009539985641)79:0.01680233951)77:0.008396922453,Genetta_maculata:0.03473924761)76:0.009900326534,Genetta_tigrina:0.04463957414)75:0.03817243455,Genetta_genetta:0.08281200869)74:0.002578230046,(Genetta_cristata:0.08057026186,(Genetta_piscivora:0.02756663851,Genetta_servalina:0.02756663851)81:0.05300362335)80:0.0048

1997688)73:0.04719441826,Genetta_johnstoni:0.132584657)72:0.02077638409,Genetta_victoriae:0.15
33610411)71:0.01900075074)69:0.03295138207,(Poiana_leightoni:0.0838238538,Poiana_richardsonii:0
.0838238538)82:0.1214893201)68:0.1634192547)62:0.002799522422)51:0.1227271074,((((((Atilax_pal
udinosus:0.1173342584,(Herpestes_naso:0.047633836,Herpestes_vitticollis:0.047633836)89:0.0697004
2244)88:0.04163029863,(((((Bdeogale_crassicauda:0.03268793739,(Bdeogale_jacksoni:0.03004163596,
Bdeogale_nigripes:0.03004163596)95:0.002646301427)94:0.0515835326,(Cynictis_penicillata:0.055546
27239,Paracynictis_selousi:0.05554627239)96:0.0287251976)93:0.01806243124,Rhynchogale_melleri:0
.1023339012)92:0.02170120284,Ichneumia_albicauda:0.1240351041)91:0.02700902254,(((Galerella_fla
vescens:0.04017469138,Galerella_ochracea:0.04017469138)99:0.06948400872,(Galerella_pulverulenta:
0.09771013697,Galerella_sanguinea:0.09771013697)100:0.01194856313)98:0.003310028152,Herpeste
s_ichneumon:0.1129687283)97:0.03807539836)90:0.007920430456)87:0.001914416815,((Herpestes_b
rachyurus:0.05132422179,Herpestes_urva:0.05132422179)102:0.0993131744,(Herpestes_edwardsi:0.0
4629111786,Herpestes_fuscus:0.04629111786,Herpestes_javanicus:0.04629111786,Herpestes_semitor
quatus:0.04629111786,Herpestes_smithii:0.04629111786)103:0.1043462783)101:0.01024157769)86:0.
00735545538,((((Crossarchus_alexandri:0.0479656539,Crossarchus_ansorgei:0.0479656539,(Crossarchu
s_obscurus:0.03812215638,Crossarchus_platycephalus:0.03812215638)108:0.009843497514)107:0.040
45697444,(Dologale_dybowskii:0.06401709523,(Helogale_hirtula:0.05328678764,Helogale_parvula:0.0
5328678764)110:0.01073030759)109:0.02440553311)106:0.01898717924,(Liberiictis_kuhni:0.1025050
804,(Mungos_gambianus:0.048717834,Mungos_mungo:0.048717834)112:0.0537872464)111:0.004904
727175)105:0.01092830063,Suricata_suricatta:0.1183381082)104:0.04989632106)85:0.1403859458,(Cr
yptoprocta_ferox:0.2383346858,((Eupleres_goudotii:0.1133615443,Fossa_fossana:0.1133615443)115:0
.09837544671,(Galidia_elegans:0.09794165974,((Galidictis_fasciata:0.0463957204,Galidictis_grandidieri
:0.0463957204)118:0.005300664519,(Mungotictis_decemlineata:0.02095359079,Salanoia_concolor:0.0
2095359079)119:0.03074279413)117:0.04624527483)116:0.1137953313)114:0.02659769475)113:0.07
028568932)84:0.1184569804,((Crocuta_crocuta:0.07910636001,(Hyaena_brunnea:0.03686690836,Hya
ena_hyaena:0.03686690836)122:0.04223945164)121:0.03958724671,Proteles_cristata:0.1186936067)1
20:0.3083837488)83:0.06718170291)50:0.2586220549)9:0.001373620413,Nandinia_binotata:0.754254
7337)8:0.07036236349,(((Ailuropoda_melanoleuca:0.2304734142,((((Helarctos_malayanus:0.06627424
506,(Ursus_americanus:0.04981236947,Ursus_thibetanus:0.04981236947)130:0.01646187559)129:0.00
3850842537,(Ursus_arctos:0.0195313319,Ursus_maritimus:0.0195313319)131:0.0505937557)128:0.02
122704757,Melursus_ursinus:0.09135213517)127:0.1167954852,Tremarctos_ornatus:0.2081476203)12
6:0.02232579387)125:0.3716428688,(((Ailurus_fulgens:0.4035130769,((((((((((((((Aonyx_capensis:0.028
13576928,Aonyx_cinerea:0.02813576928)149:0.004427230216,Lutrogale_perspicillata:0.0325629995)1
48:0.007574990495,((Lutra_lutra:0.016774676,Lutra_nippon:0.016774676)151:0.01092082001,Lutra_s
umatrana:0.02769549601)150:0.01244249398)147:0.007229600349,Hydrictis_maculicollis:0.047367590
34)146:0.00252544187,(Lontra_canadensis:0.02804048557,((Lontra_felina:0.005166298303,Lontra_pro
vocax:0.005166298303)154:0.005285346812,Lontra_longicaudis:0.01045164512)153:0.01758884046)1
52:0.02185254664)145:0.05716513841,Enhydra_lutris:0.1070581706)144:0.002550356563,(Galictis_cuj
a:0.03344223706,Galictis_vittata:0.03344223706)155:0.07616629013,Ictonyx_libyca:0.1096085272,Poe
cilogale_albinucha:0.1096085272,Pteronura_brasiliensis:0.1096085272)143:0.02055254804,((((Mustela
_africana:0.02317562805,Mustela_felipei:0.02317562805)159:0.0152301946,Mustela_frenata:0.03840
582265)158:0.008795901654,(Neovison_macrodon:0.02451109646,Neovison_vison:0.02451109646)16
0:0.02269062785)157:0.07604083504,((((Mustela_altaica:0.0396673154,(Mustela_nivalis:0.020578541
59,Mustela_subpalmata:0.02057854159)165:0.01908877381)164:0.0167087275,(((Mustela_eversmanii:

0.01396343983,Mustela_putorius:0.01396343983)168:0.005854979379,Mustela_lutreola:0.019818419
21,Mustela_lutreolina:0.01981841921,Mustela_nigripes:0.01981841921,(Mustela_nudipes:0.01650741
44,Mustela_strigidorsa:0.0165074144)169:0.003311004816,Mustela_sibirica:0.01981841921)167:0.018
84020652,Mustela_itatsi:0.03865862573)166:0.01771741717)163:0.01421159937,Mustela_erminea:0.0
7058764227)162:0.01030222806,Mustela_kathiah:0.08088987034)161:0.04235268901)156:0.00691851
5883)142:0.005436516743,(Ictonyx_striatus:0.05265822638,Vormela_peregusna:0.05265822638)170:0.
08293936559)141:0.01941667228,Melogale_moschata:0.1550142643)140:0.0078496783,((Eira_barbar
a:0.1073587683,Martes_pennanti:0.1073587683)172:0.003200491791,(Gulo_gulo:0.1031044337,((Mar
tes_americana:0.02713814692,(Martes_foina:0.02485386445,((Martes_martes:0.007325139645,Marte
s_zibellina:0.007325139645)178:0.002121814199,Martes_melampus:0.009446953844)177:0.01540691
061)176:0.00228428247)175:0.0435087683,(Martes_flavigula:0.02931381861,Martes_gwatkinsii:0.029
31381861)179:0.04133309661)174:0.03245751849)173:0.00745482639)171:0.05230468245)139:0.023
22587214,((Arctonyx_collaris:0.04300546151,((Meles_anakuma:0.02570179853,Meles_leucurus:0.0257
0179853)183:0.01406570641,Meles_meles:0.03976750494)182:0.003237956569)181:0.05275574681,(
Melogale_everetti:0.05583179162,Melogale_orientalis:0.05583179162,Melogale_personata:0.0558317
9162)184:0.03992941669)180:0.09032860637)138:0.04015643524,Taxidea_taxus:0.2262462499)137:0.
1125133144,(Lyncodon_patagonicus:0.05881222801,Mellivora_capensis:0.05881222801)185:0.279947
3363)136:0.01959143985,((Bassaricyon_alleni:0.2197054303,Bassaricyon_beddardi:0.2197054303,Bass
aricyon_gabbii:0.2197054303,Bassaricyon_lasius:0.2197054303,Bassaricyon_pauli:0.2197054303,(Bassa
riscus_astutus:0.1155274284,Bassariscus_sumichrasti:0.1155274284)188:0.1041780018,((Nasua_narica:
0.05532403815,Nasua_nasua:0.05532403815)190:0.06634631331,Nasuella_olivacea:0.1216703515)189
:0.0980350788,(Procyon_cancrivorus:0.05788509116,Procyon_lotor:0.05788509116,Procyon_pygmaeu
s:0.05788509116)191:0.1618203391)187:0.06415377709,Potos_flavus:0.2838592074)186:0.074491796
82)135:0.04516207276)134:0.009887260011,((((Conepatus_chinga:0.05539381981,Conepatus_leucono
tus:0.05539381981)195:0.06128844244,(Conepatus_humboldtii:0.05534141512,Conepatus_semistriatu
s:0.05534141512)196:0.06134084713)194:0.05566607002,((Mephitis_macroura:0.03637027662,Mephit
is_mephitis:0.03637027662)198:0.08158983328,((Spilogale_angustifrons:0.03411459064,Spilogale_put
orius:0.03411459064)200:0.0022078977,Spilogale_gracilis:0.03632248834,Spilogale_pygmaea:0.036322
48834)199:0.08163762156)197:0.05438822236)193:0.05820192111,(Mydaus_javanensis:0.0709700449
,Mydaus_marchei:0.0709700449)201:0.1595802085)192:0.1828500836)133:0.1647892721,((((((((Arcto
cephalus_australis:0.02532947328,Arctocephalus_galapagoensis:0.02532947328)210:0.008224767329,
Arctocephalus_forsteri:0.03355424061)209:0.04543270416,((Arctocephalus_gazella:0.00307179257,Arc
tocephalus_tropicalis:0.00307179257)212:0.07364801339,(Arctocephalus_philippii:0.01133993725,Arct
ocephalus_townsendi:0.01133993725)213:0.06537986871)211:0.00226713881)208:0.01798660542,(Ne
ophoca_cinerea:0.08234832606,Phocarctos_hookeri:0.08234832606)214:0.01462522413)207:0.004661
04329,(Arctocephalus_pusillus:0.09913063553,Otaria_flavescens:0.09913063553)215:0.002503957951)
206:0.01396151995,(Eumetopias_jubatus:0.08669628223,((Zalophus_californianus:0.00285440515,Zalo
phus_wollebaeki:0.00285440515)218:0.06133597687,Zalophus_japonicus:0.06419038202)217:0.02250
590021)216:0.02889983121)205:0.03145641477,Callorhinus_ursinus:0.1470525282)204:0.1389017403,
Odobenus_rosmarus:0.2859542685)203:0.02192990635,(((Cystophora_cristata:0.09392895138,(((((Hali
choerus_grypus:0.02181322133,Pusa_caspica:0.02181322133)226:0.007742458432,Pusa_sibirica:0.029
55567976)225:0.001633830017,Pusa_hispida:0.03118950978)224:0.005842454859,(Phoca_largha:0.01
922735167,Phoca_vitulina:0.01922735167)227:0.01780461296)223:0.03808298175,(Histriophoca_fasci
ata:0.05643137175,Pagophilus_groenlandicus:0.05643137175)228:0.01868357464)222:0.01881400499)

221:0.06267982747,Erignathus_barbatus:0.1566087788)220:0.05074036488,(((((Hydrurga_leptonyx:0.0
395577549,Leptonychotes_weddellii:0.0395577549)233:0.03890062594,Ommatophoca_rossii:0.078458
38084)232:0.001496908784,Lobodon_carcinophaga:0.07995528963)231:0.04814865817,(Mirounga_an
gustirostris:0.02935753617,Mirounga_leonina:0.02935753617)234:0.09874641162)230:0.01456671115
,(Monachus_monachus:0.1199588243,(Monachus_schauinslandi:0.05571257023,Monachus_tropicalis:0
.05571257023)236:0.0642462541)235:0.02271183462)229:0.06467848478)219:0.1005350311)202:0.27
03054341)132:0.02392667398)124:0.1887702136,(((((((Atelocynus_microtis:0.1032544267,((Dusicyon_
australis:0.03242670062,Vulpes_ferrilata:0.03242670062)245:0.06680440401,(((Lycalopex_culpaeus:0.
03371079869,Lycalopex_sechurae:0.03371079869)248:0.003398992835,(Lycalopex_griseus:0.03367937
155,Lycalopex_gymnocercus:0.03367937155)249:0.003430419975)247:0.01208083182,(Lycalopex_fulvi
pes:0.01247309444,Lycalopex_vetulus:0.01247309444)250:0.0367175289)246:0.05004048129)244:0.00
4023322061)243:0.009772301895,Cerdocyon_thous:0.1130267286)242:0.09175492383,Chrysocyon_br
achyurus:0.2047816524)241:0.002571904889,((Canis_adustus:0.09787178703,Canis_mesomelas:0.097
87178703)252:0.1075992569,((((Canis_aureus:0.05940149622,Canis_latrans:0.05940149622)256:0.005
668398719,Canis_lupus:0.06506989493)255:0.03644428586,Canis_simensis:0.1015141808)254:0.00459
7356449,Cuon_alpinus:0.1061115372)253:0.09935950669)251:0.001882513381)240:0.003546302029,(
Lycaon_pictus:0.1586869697,Speothos_venaticus:0.1586869697)257:0.05221288962)239:0.087333497
51,((Nyctereutes_procyonoides:0.2918880464,(((((Vulpes_bengalensis:0.03084810057,Vulpes_corsac:0.
03084810057)264:0.03331774906,(Vulpes_rueppellii:0.02684846411,Vulpes_vulpes:0.02684846411)26
5:0.03731738553)263:0.0155771947,((Vulpes_lagopus:0.03259066279,Vulpes_macrotis:0.03259066279
)267:0.008787486661,Vulpes_velox:0.04137814945)266:0.03836489488)262:0.01970820081,(Vulpes_c
ana:0.05126906434,Vulpes_zerda:0.05126906434)268:0.0481821808)261:0.02506143548,(Vulpes_cha
ma:0.05795389865,Vulpes_pallida:0.05795389865)269:0.06655878196)260:0.1673753658)259:0.00302
9858266,Otocyon_megalotis:0.2949179047)258:0.00331545218)238:0.002813963125,(Urocyon_cinere
oargenteus:0.02810075944,Urocyon_littoralis:0.02810075944)270:0.2729465605)237:0.4898391766)12
3:0.03373060061)7:0.1145378488)5:0.0008474735539,((Artibeus_jamaicensis:0.4064365375,Mystacina
_tuberculata:0.4064365375)272:0.1207712226,Tadarida_brasiliensis:0.5272077601)271:0.4127946595)
4:0.0599975804)1;

Gymnotiform Phylogeny

((Electrophorus_electricus_39371:72.23241487,((Gymnotus_pantherinus_11144:4.495935398,Gymnotu
s_capitimaculatus:4.495935398):32.41810672,((((Gymnotus_melanopleura:1.692596401,(Gymnotus_on
ca:0.6772868529,Gymnotus_jonasi_34047:0.6772868529):1.015309548):1.035510705,Gymnotus_steno
leucus_GQ862680:2.728107106):9.648416954,(Gymnotus_coropinae_43746:6.961479619,(Gymnotus_c
oatesi_GQ862657:2.250129115,Gymnotus_javari_GQ862670:2.250129115):4.711350503):5.415044441
):21.16192851,(((Gymnotus_cataniapo_GQ862655:5.518516643,Gymnotus_tiquie:5.518516643):13.984
90392,(Gymnotus_cf_anguillaris_NRL_2009:15.49235907,Gymnotus_pedanopterus_GQ862678:15.4923
5907):4.011061497):11.26565773,((((Gymnotus_tigre_060406:12.52090957,(Gymnotus_esmeraldas:4.1
36529317,Gymnotus_henni_8231:4.136529317):8.384380255):3.188463037,Gymnotus_paraguensis:15.
70937261):4.870910914,Gymnotus_inaequilabiatus:20.58028352):5.713853776,((Gymnotus_panamens
is_8021:9.016015791,(Gymnotus_maculosus_8126:1.987403447,Gymnotus_cylindricus_1201:1.987403
447):7.028612344):9.506317752,(Gymnotus_diamantinensis:15.18021361,((Gymnotus_varzea_GQ8626
87:7.909177679,(Gymnotus_pantanal_32017:5.971380305,(Gymnotus_obscurus_GQ862675:4.3805417
56,Gymnotus_curupira_GQ862665:4.380541756):1.590838549):1.937797374):4.43444342,((((Gymnotu

s_omarorum_7093:2.744066688,Gymnotus_chimarrao:2.744066688):1.844204535,Gymnotus_sylvius_3
6021:4.588271223):0.8391557308,Gymnotus_mamiraua_GQ862673:5.427426954):1.445764719,((Gym
notus_chaviro_39364:2.935083766,(Gymnotus_ardilai_8175:1.191646991,Gymnotus_choco_8209:1.19
1646991):1.743436775):1.933726012,(Gymnotus_capanema:2.606551585,(Gymnotus_carapo_35859:0.
9405526706,(Gymnotus_bahianus_7245:0.6485245388,(Gymnotus_arapaima_GQ862647:0.341992334
7,Gymnotus_ucamara_GQ862685:0.3419923347):0.3065322041):0.2920281318):1.665998914):2.26225
8193):2.004381895):5.470429427):2.836592512):3.342119932):7.771803756):4.474940996):2.7693742
75):3.375589544):35.31837276):10.54584971,((((((Microsternarchus_bilineatus_50417:11.82440828,Pr
ocerusternarchus_pixuna:11.82440828):13.45648863,Racenisia_fimbriipinna_gi_01:25.28089691):11.63
179591,Hypopomus_artedi_GQ862689:36.91269282):2.605071787,(((((Brachyhypopomus_draco_1626
7:3.51057973,Brachyhypopomus_beebei_39375:3.51057973):9.372808747,(Brachyhypopomus_gauderi
o:4.390675692,Brachyhypopomus_pinnicaudatus_gi_01:4.390675692):8.492712785):3.90965006,(Brac
hyhypopomus_walteri:4.779530717,Brachyhypopomus_bennetti:4.779530717):12.01350782):5.253963
423,(Brachyhypopomus_janeiroensis:5.386209221,Brachyhypopomus_jureiae:5.386209221):16.660792
74):5.579167316,((Brachyhypopomus_brevirostris_GQ:17.95654411,Brachyhypopomus_bullocki_Sulliva
n:17.95654411):6.017266268,(Brachyhypopomus_bombilla:19.33755355,(Brachyhypopomus_occidenta
lis_1849:6.617654576,Brachyhypopomus_diazi_GQ862642:6.617654576):12.71989897):4.636256826):3
.652358902):11.89159533):10.74560047,Akawaio_penak:50.26336508):16.54345636,((((Steatogenys_el
egans_182571:5.848276207,Steatogenys_ocellatus:5.848276207):15.76536646,Steatogenys_duidae_34
068:21.61364267):23.20840539,(Hypopygus_neblinae_14841:24.94905175,(Hypopygus_isbruckeri:17.9
5236467,((Hypopygus_nijsseni:3.750362948,Hypopygus_ortegai:3.750362948):9.007555267,(Hypopygu
s_cryptogenys:8.299905645,(Hypopygus_lepturus_43739:5.114990754,(Hypopygus_benoneae:2.85068
5675,(Hypopygus_minissimus:1.06493186,Hypopygus_hoedemani:1.06493186):1.785753815):2.264305
079):3.184914891):4.458012571):5.194446453):6.996687083):19.87299631):12.15628688,((((Gymnorh
amphichthys_rosamariae_191142:11.44086191,Gymnorhamphichthys_hypostomus_18063:11.4408619
1):8.779311428,Gymnorhamphichthys_britskii_22012:20.22017334):1.288790209,(Gymnorhamphichth
ys_bogardusi_191143:9.946991461,(Gymnorhamphichthys_petiti_179685:9.587626134,Gymnorhamphi
chthys_rondoni_179673:9.587626134):0.3593653268):11.56197209):23.53610139,(Iracema_caiana:21.
92740804,((Rhamphichthys_lineatus_116566:7.806061319,(Rhamphichthys_hahni_19226:1.275447983,
Rhamphichthys_drepanium_Sullivan:1.275447983):6.530613336):3.752525557,(Rhamphichthys_apuren
sis_43111:7.983478433,(Rhamphichthys_rostratus_187120:5.180994397,(Rhamphichthys_atlanticus:3.
071449357,Rhamphichthys_marmoratus_42545:3.071449357):2.10954504):2.802484035):3.575108443
):10.36882117):23.11765689):11.93327):9.828486502):12.5371097,((((((Sternopygus_obtusirostris:4.81
4036312,Sternopygus_macrurus_39502:4.814036312):12.29136609,(Sternopygus_xingu_19643:14.004
60746,(Sternopygus_arenatus:8.692861269,(Sternopygus_pejeraton:4.242494579,(Sternopygus_darien
sis_604059707:1.400389576,Sternopygus_aequilabiatus:1.400389576):2.842105003):4.45036669):5.31
1746194):3.100794943):5.836816912,Sternopygus_branco:22.94221932):14.16535842,Sternopygus_ast
rabes:37.10757774):31.21357171,(((Distocyclus_conirostris_182573:20.11674574,(Archolaemus_orient
alis:14.0124569,(((Archolaemus_ferreirai:1.543659491,Archolaemus_luciae:1.543659491):3.032535137,
Archolaemus_santosi:4.576194627):4.356040395,(Archolaemus_blax_AF072163:2.718999423,Archolae
mus_janeae:2.718999423):6.213235599):5.080221877):6.104288838):5.415934901,(Distocyclus_goajira
:21.99586311,(((Eigenmannia_humboldtii:11.19481086,(Eigenmannia_nigra:3.722741095,Eigenmannia_
cf_limbata_189019:3.722741095):7.472069765):3.191755091,Eigenmannia_trilineata_36072:14.386565
95):4.400741085,((Eigenmannia_macrops_37145:3.521098565,Japigny_kirschbaum:3.521098565):11.57

254474,(Eigenmannia_microstoma_4254:10.10564014,(Eigenmannia_virencens_45735:6.30574794,Eig
enmannia_vicentespelaea_62040:6.30574794):3.799892196):4.98800317):3.693663731):3.208556075):
3.536817527):10.25643669,((Rhabdolichops_lundbergi:6.045273306,Rhabdolichops_nigrimans:6.04527
3306):19.67965237,(Rhabdolichops_zareti:17.82638324,((Rhabdolichops_eastwardi:4.020626069,Rhab
dolichops_caviceps:4.020626069):8.821231347,(((Rhabdolichops_electrogrammus:1.791437429,Rhabd
olichops_jegui_189017:1.791437429):3.45995717,Rhabdolichops_navalha:5.251394599):4.157777445,(
Rhabdolichops_cf_stewarti_49295:3.348091428,Rhabdolichops_troscheli:3.348091428):6.061080616):3
.432685372):4.984525821):7.898542441):10.06419165):32.53203212):6.839693217,((Orthosternarchus
_tamandua_U15235:11.53619755,Sternarchorhamphus_muelleri_182579:11.53619755):33.37895427,((
Adontosternarchus_balaenops_182572:15.35238829,(Adontosternarchus_devenanzii_19126:10.823696
62,((Adontosternarchus_sachsi_188863:3.160037025,Adontosternarchus_duartei:3.160037025):5.9496
1562,(Adontosternarchus_clarkae_182580:1.023886658,Adontosternarchus_nebulosus_14826:1.02388
6658):8.085765987):1.714043979):4.528691668):17.08748978,((Parapteronotus_hasemani_178360:22.
52540116,((Megadonognathus_cuyuniense:5.449329491,Megadontognathus_kaitukaensis:5.44932949
1):15.76520803,(((Apteronotus_acidops:3.242143527,Apteronotus_brasiliensis:3.242143527):8.338028
273,(Sternarchella_curvioperculata:3.290399389,Tembeassu_marauna:3.290399389):8.28977241):8.28
1378157,((((((Apteronotus_leptorhynchus_190772:2.211484342,Apteronotus_lindalvae:2.211484342):3
.976881219,(Apteronotus_ferrarisi:3.453005281,(Apteronotus_galvisi:1.225414823,Apteronotus_macro
stomus:1.225414823):2.227590458):2.73536028):2.868274611,(Apteronotus_pemon:2.958939856,Apte
ronotus_baniwa:2.958939856):6.097700315):3.123466256,(Apteronotus_spurrellii:6.195596979,(Apter
onotus_anu:2.072603502,Apteronotus_rostratus:2.072603502):4.122993477):5.984509448):3.1129211
3,(Apteronotus_magdalenensis:4.61701134,Apteronotus_cuchillo:4.61701134):10.67601622):3.253397
686,((((Apteronotus_eschmeyeri:3.991103817,(Apteronotus_milesi:1.417002824,Apteronotus_mariae:1
.417002824):2.574100993):3.759574036,Apteronotus_jurubidae:7.750677853):4.469452204,Apteronot
us_magoi:12.22013006):3.431820081,(Apteronotus_cuchillejo:11.45407487,(Apteronotus_albifrons_16
150:8.280890623,(Apteronotus_camposdapazi:3.137237301,Apteronotus_caudimaculosus_43246:3.13
7237301):5.143653323):3.17318425):4.197875265):2.894475104):1.315124714):1.352987561):1.31086
3642):4.455033365,(((((Compsaraia_samueli_182210:3.956088511,Compsaraia_compsus:3.956088511):
10.81516832,((Porotergus_duende:4.144657429,(Porotergus_gimbelli_178277:1.52471599,Porotergus_
gymnotus:1.52471599):2.619941439):2.944982714,(Apteronotus_apurensis:4.650555715,(Apteronotus
_macrolepis:2.703842284,(Apteronotus_bonapartii_37171:1.440256681,Apteronotus_ellisi_24040:1.44
0256681):1.263585603):1.946713431):2.439084428):7.681616688):1.520608967,(Sternarchogiton_porc
inum_182212:13.41859374,(Sternarchogiton_nattereri_37136:5.039297747,(Sternarchogiton_preto_57
528:1.121495356,(Sternarchogiton_zuanoni:0.4829220276,Sternarchogiton_labiatus_189003:0.482922
0276):0.638573328):3.917802391):8.379295994):2.873272057):2.03485445,(Pariosternarchus_amazon
ensis:15.00300697,((Sternarchella_sima:9.651187365,Sternarchella_orinoco:9.651187365):2.42247877
3,((Sternarchella_calhamazon_46987:0.9516804697,(Sternarchella_orthos:0.4125883654,Sternarchella_
terminalis_182576:0.4125883654):0.5390921043):6.747374016,(Sternarchella_schotti:3.941518144,(M
agosternarchus_raptor:1.374299657,Magosternarchus_duccis:1.374299657):2.567218488):3.75753634
2):4.374611652):2.929340829):3.32371328):5.348512979,((Platyurosternarchus_crypticus_179153:1.49
8783581,Platyurosternarchus_macrostomus_18:1.498783581):20.79937023,(((Sternarchorhynchus_goe
ldii:3.03547271,Sternarchorhynchus_oxyrhynchus:3.03547271):7.6262545,(Sternarchorhynchus_axelro
di:2.796746227,Sternarchorhynchus_mormyrus_182583:2.796746227):7.864980984):6.972766976,((Ste
rnarchorhynchus_curumim:4.615558016,Sternarchorhynchus_caboclo:4.615558016):11.74890431,(Ster

narchorhynchus_severii:15.20834134,((Sternarchorhynchus_inpai:4.310218433,Sternarchorhynchus_m
ontanus:4.310218433):9.786917092,(Sternarchorhynchus_britskii:13.08773639,((Sternarchorhynchus_g
nomus:3.935448835,Sternarchorhynchus_marreroi:3.935448835):8.182346636,((Sternarchorhynchus_
mareikeae:3.748601611,Sternarchorhynchus_villasboasi:3.748601611):7.447354552,((Sternarchorhync
hus_curvirostris:5.573607489,(Sternarchorhynchus_schwassmanni:1.785103922,Sternarchorhynchus_st
arski_47080:1.785103922):3.788503566):4.790618137,((Sternarchorhynchus_hagedornae_36892:5.540
985176,(Sternarchorhynchus_freemani:2.2773229,Sternarchorhynchus_galibi_187155:2.2773229):3.26
3662276):3.560251403,((((Sternarchorhynchus_cramptoni:1.164101466,Sternarchorhynchus_stewarti:1
.164101466):2.017831286,Sternarchorhynchus_retzeri:3.181932752):2.444557371,(Sternarchorhynchus
_taphorni:2.071178943,Sternarchorhynchus_yepezi:2.071178943):3.555311181):2.29164735,((Sternarc
horhynchus_chaoi:3.547081161,(Sternarchorhynchus_kokraimoro:1.267974315,Sternarchorhynchus_jai
mei:1.267974315):2.279106846):2.761160276,((Sternarchorhynchus_roseni:2.413592055,(Sternarchorh
ynchus_higuchii:0.9078286134,Sternarchorhynchus_mendesi:0.9078286134):1.505763442):1.82146397
8,Sternarchorhynchus_mesensis:4.235056033):2.073185404):1.609896036):1.183099105):1.262989047
):0.8317305378):0.9218393073):0.9699409146):1.009399139):1.111205812):1.156120992):1.27003185
7):4.663659624):1.377079416):3.305201299):5.459443544):12.47527375):30.24569085):4.183088469):
3.434333442):68.4541427;
